# Moving to Capture Children’s Attention: Developing a Methodology for Measuring Visuomotor Attention

**DOI:** 10.1371/journal.pone.0159543

**Published:** 2016-07-19

**Authors:** Liam J. B. Hill, Rachel O. Coats, Faisal Mushtaq, Justin H. G. Williams, Lorna S. Aucott, Mark Mon-Williams

**Affiliations:** 1 School of Psychology, University of Leeds, Leeds, West Yorkshire, United Kingdom; 2 Institute of Medical Sciences, University of Aberdeen, Aberdeen, Scotland, United Kingdom; Tokai University, JAPAN

## Abstract

Attention underpins many activities integral to a child’s development. However, methodological limitations currently make large-scale assessment of children’s attentional skill impractical, costly and lacking in ecological validity. Consequently we developed a measure of ‘Visual Motor Attention’ (VMA)—a construct defined as the ability to sustain and adapt visuomotor behaviour in response to task-relevant visual information. In a series of experiments, we evaluated the capability of our method to measure attentional processes and their contributions in guiding visuomotor behaviour. Experiment 1 established the method’s core features (ability to track stimuli moving on a tablet-computer screen with a hand-held stylus) and demonstrated its sensitivity to principled manipulations in adults’ attentional load. Experiment 2 standardised a format suitable for use with children and showed construct validity by capturing developmental changes in executive attention processes. Experiment 3 tested the hypothesis that children with and without coordination difficulties would show qualitatively different response patterns, finding an interaction between the cognitive and motor factors underpinning responses. Experiment 4 identified associations between VMA performance and existing standardised attention assessments and thereby confirmed convergent validity. These results establish a novel approach to measuring childhood attention that can produce meaningful functional assessments that capture how attention operates in an ecologically valid context (i.e. attention's specific contribution to visuomanual action).

## Introduction

Measuring ‘attention’ in educational settings is fundamentally important because of the integral role that this cognitive faculty plays in determining academic success. Epidemiological studies report that attention skills at 4–6 years old predict: maths and reading ability throughout school [1], overall academic attainment at 13 years old [2] and whether an individual has graduated college by the age of 25 years [3]. This concurs with clinical research showing attention deficit hyperactivity disorder (ADHD) is a risk factor for impaired academic achievement [4,5]. It follows, therefore, that regular objective assessment of children’s attentional abilities throughout their mainstream education may be beneficial [6], facilitating more timely identification of those in need of additional support. However, there are both applied and theoretical challenges that need to be overcome before widespread in-school assessment becomes viable.

Practically, many traditional standardised methodologies for assessing attention are too time consuming or costly to be carried out on a large scale (e.g. 1-to-1 diagnostic interviews, pencil-and-paper psychometric tests, classroom observation techniques). Some of these methods are also highly susceptible to assessor bias [7]. Meanwhile, brief parent and/or teacher report questionnaires are useful as multi-informant viewpoints on a child’s behaviour but are too subjective to be trusted as reliable and accurate measurements in isolation [8,9]. Computerised psychometric tests offer a potentially promising alternative [10] due to their digitised format enabling improved objectivity and reliability and cost efficiency—making them amenable for use in population-based epidemiological studies [11,12]. Consequently, a number of standardised computerised assessments have been developed to measure children’s attention skills (e.g. [13,14–17]). However, there are several common limitations in the methodologies such electronic assessments use, which undermine their construct validity.

Many tests focus on obtaining point estimate measures of attentive capacity (e.g. mean response time, total number of responses correct) [18] despite growing evidence that intra-individual variability (IIV) across time is a more appropriate metric for capturing *sustained* attention [19,20]. Indeed, adults [21] and children [22,23] with attention difficulties produce qualitatively different variability patterns compared to controls in laboratory based serial reaction time tasks, while meta-analyses suggest IIV is a core characteristic of ADHD [24]. In addition, dynamic interactions between perception, action and cognition are widely acknowledged [25–28] but this complexity is often overlooked by standardised psychometric assessments. Instead, many tasks classify visuomotor responses as unambiguous indices of cognitive functions [29,30]. Thus new methodologies ideally need developed that enable participants’ responses to be understood as an *interaction* between cognitive abilities and task-dependent perceptuomotor influences. This more nuanced perspective would be valuable in light of the evidence, for example, for there being systematic differences between how we cognitively process information about objects within versus beyond our grasp (for a review see [31]).

The often arbitrary delineation between cognitive and motor behaviours is illustrated in studies that employ manual tracking tasks as concurrent ‘distractor’ sub-tasks within dual-task methodologies [32–35]. Such methodologies view each sub-task as acting in direct competition for limited cognitive resources. Thus they implicitly acknowledge visuomotor control as an attentionally demanding activity and, moreover, a process capable of moderating an individual’s ability to simultaneously perform more abstract cognitive tasks. This observation led us to develop a novel computerised task to assess children’s attention skills over time; and the relationship between those skills and visuomotor control.

The task assesses core sub-constructs within the greater ‘attentional network’ [16] in recognition of the fact that attention is a complex multidimensional construct [36–38], which is too broad to be amenable to direct empirical measurement [18]. We focussed on sustained attention (how well an individual maintains performance on a task over an extended period of time [36]) and executive attention (how successfully an individual can monitor and control distribution of their limited attentional resources [39]). Executive attention is integral to performance of the following functions: (i) holding goal relevant stimuli/instructions in mind; (ii) eliciting inhibitory control over goal-irrelevant responses; and (iii) directing conscious focus toward goal-relevant stimuli in the presence of competing distractors [40].

Our manual tracking task required participants to track targets on a tablet laptop using a handheld stylus (a ‘pen-to-paper’ type interface) and included subsets of trials assessing Sustained and Executive aspects of Attention respectively. The latter required participants to concurrently track whilst also attending to visual information presented in a different spatial location (information which periodically directed them to change their tracking behaviour). An intentional, strength of this approach is that it converges with an analysis of the concurrent attentional and motor demands faced by children within the classroom. A classic example of this is the need for a child to solve problems at their desk whilst also processing visual information presented on a whiteboard at the front of the classroom.

This paper presents four experiments that describe the development of this innovative method and its validation as a measure of sustained and executive attention, captured via motor performance. The construct under consideration is therefore hereafter described as ‘VisuoMotor Attention’ (VMA).

## Experiment 1

### Introduction

Existing standardised computerised assessments of sustained and/or executive aspects of Attention [13–16] typically examine these sub-constructs using relatively abstract response methods (e.g. response key/button presses) and summarise attentive functioning as a measure of central tendency within these responses (e.g. mean reaction time). Separately, visuo-manual tracking has historically been classified in the literature as a measure of motor coordination and been considered dissociable from higher-order cognitive functioning [41–43]. Such definitions are undermined though by more recent experimental research that has provided uniquely detailed insights into the continuity of attentional processes and their relationships with action [34].

The basic attentional demands of tracking a single moving target manually are approximately equivalent to those required from a traditional ‘sustained vigilance’ continuous performance task—the most widely used methodology for assessing sustained attention [17]. Advantageously though, the continuous response required during visuomotor tracking enables a far more detailed record of performance across time (only limited by equipment sampling rate). In dual-task scenarios, when further cognitive demands are presented in competition, the continuous nature of this recording also enables a more nuanced analysis of precisely how a participant’s limited attentional resources are executively managed across tasks. For example, Boiteau et al. [34] asked participants to manually track whilst concurrently engaged in a verbal conversation and observed tracking performance deteriorating where the competing conversation demanded increased attentiveness. This capacity to detect brief temporary shifts in focus of attention is a unique advantage that continuous visuomotor assessments hold over traditional serial-repeating trial methodologies.

Assessments that integrate complex visuomotor behaviour with cognitive demands have also been proposed to be particularly ecologically relevant, because of their functional similarity to a variety of important everyday behaviours. For example, talking during driving [34] or walking [44]; executing any motoric behaviour (e.g. writing) whilst concurrently cognitively ‘composing’ the symbolic intention(s) underlying that action [45]. Experiment 1 therefore established a specific methodology based around visuomotor behaviour and confirmed its sensitivity to attentional manipulation under a series of single- and dual-task conditions. Dual-task conditions included a further ‘target-switching’ component in addition to manual tracking, which required participants to serially track one of four on-screen targets continuously and periodically switch their tracking behaviour between these targets in response to cues presented on the periphery of the computer screen.

We tested the hypothesis that altering cue-salience within the secondary cue-detection task (a manipulation of competing attentional load) would alter performance in a predictable manner on both this task and the concurrent tracking task. Applying the principal of ‘limited processing capacity’ [46], participants’ tracking performance was expected to be poorer under dual-task conditions compared to an undistracted (single-task) tracking condition. Feature Integration Theory [47] also predicted that dual-task conditions in which ‘single-feature’ cues were presented would induce a lower competing attentional demand than those in which ‘combined-feature’ cues were presented, due to the additional cognitive effort required to ‘feature-bind’ multiple characteristics [48]. If task performance varied systematically in response to such manipulations of attentional load this would support the use of this novel methodology and challenge existing descriptions of visuomanual tracking as being cognitively inconsequential [41–43].

### Method

#### Ethics statement

For all of the experiments presented in this paper, ethical approval was obtained from either the University of Leeds ethics committee and/or the University of Aberdeen College Ethics Review Board, dependent on whether the study recruited participants in one or both locations. In experiments where adults were tested they gave their own informed written consent to participate. For children, the informed written consent of both the participating child and their parent/guardian was obtained.

#### Participants

Forty-nine self-reportedly healthy adults (25 male, 24 female; median age [range] = 30 years [20 to 50]) with normal or corrected-to-normal vision were recruited through informal networks.

#### Materials

The Visuomotor Attention (VMA) task was designed in and presented using custom-built software capable of presenting visual stimuli whilst simultaneously recording participants’ kinematic responses via a hand-held stylus [49]. Deployed on a Toshiba Model Tecra M7 tablet portable computer (screen: 303 x 109 mm, 1600 x 1200 pixels, 16-bit colour, 60 Hz refresh rate), the screen digitiser measured planar position of the stylus at a rate of 120 Hz, allowing precise measurements of complex movement to be reliably captured.

#### Procedure

Participants sat at a desk in a quiet room with the tablet computer in front of them. The tablet screen was displayed horizontally in front of the participant and they interacted with the screen using a stylus held in their preferred hand. In a single session lasting approximately 45 minutes they completed four conditions, one ‘single-target’ condition and three different ‘cue-detection’ conditions (entitled: colour, shape and combined). Each condition was 8 minutes in duration (4 x 2 min trials) and their order of presentation was counterbalanced between participants. Prior to completing these conditions participants received a set of pre-recorded instructions and undertook four practice trials (1 per condition, each 1 min long).

**Fig 1:**
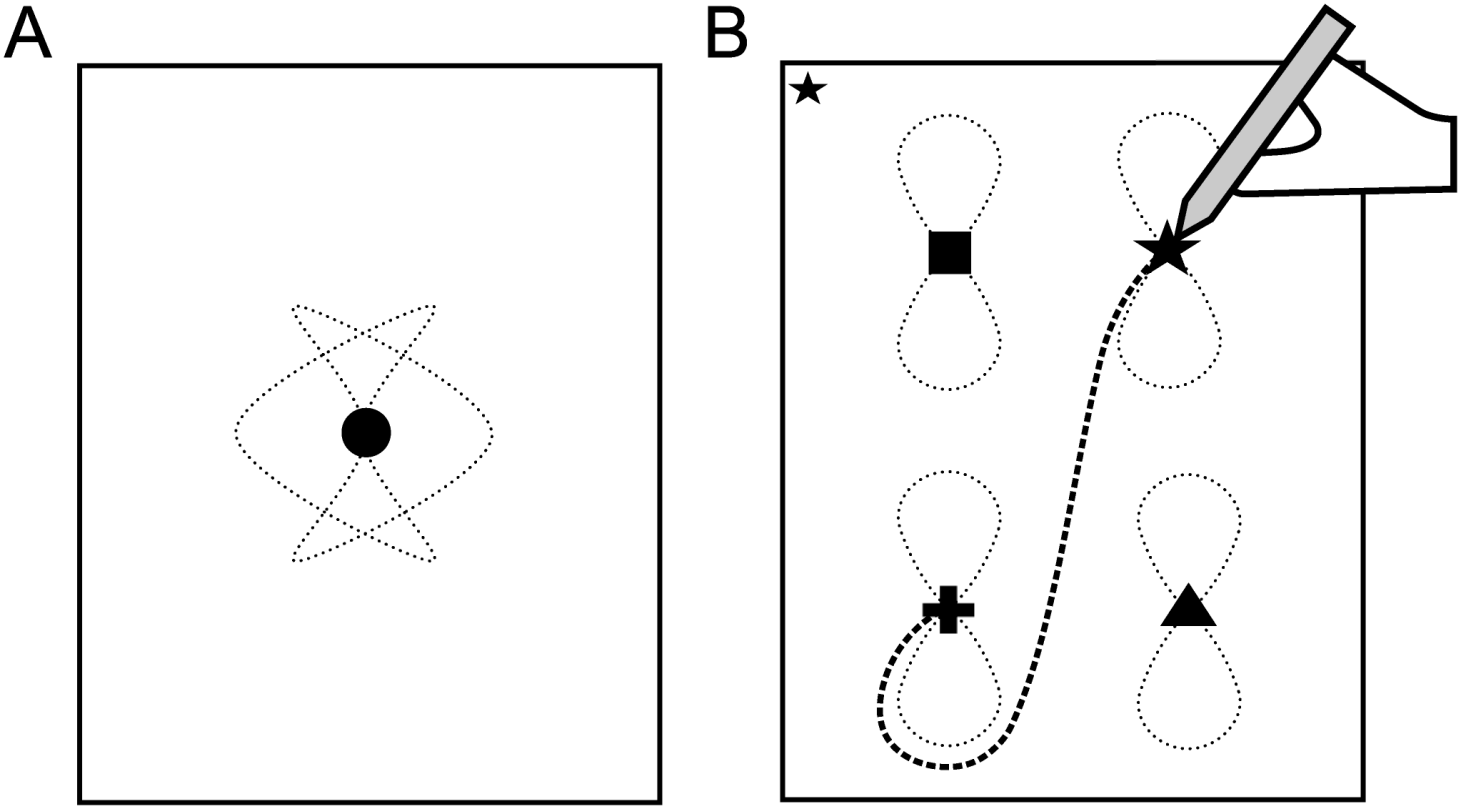
Schematic of the Visual Motor Attention (VMA) Task. An illustration of the on-screen stimuli and participant’s interactions with them during (A) a single-target and (B) a shape trial—two of the four experimental conditions used in Experiment 1. On-screen movement of targets is illustrated using dotted lines, with examples of the ‘Figure-8’ and ‘Boomerang’ paths presented here (1 per panel). Participant’s movement of the stylus is illustrated with a bold-dashed line and in the shape trial (Panel B) illustrates a period of time within this trial during which a valid cue is presented and the participant responds correctly to it by relocating their tracking behaviour from one target to another.

*Single-target condition*: Participants began a trial by placing the stylus on a stationary dot in the centre of an otherwise blank screen. Following a two second delay the dot began to move around the screen and participants were required to follow it using their stylus, trying to stay as close to its centre for the remainder of the trial. The movement pattern comprised forty consecutive ‘paths’ presented in a pseudo-random order. Each path was one of two different shapes (“Figure-8” or “Boomerang”, see Fig 1), generated through coupled sinusoidal oscillations of the dot along its horizontal and vertical axes at different relative amplitudes and frequencies. Each path also varied between one of two different sizes (“Small” paths were two-thirds the amplitude of “Large” paths). Cumulatively this amounted to four different path types (i.e. “Small Figure-8”, “Large Figure-8”, “Small Boomerang” and “Large Boomerang), each presented 10 times per trial.

*Cue-detection conditions*: All three cue-detection conditions (colour, shape and combined) used the same basic screen layout; which comprised of four targets (as opposed to one), with each presented in a different quadrant of the screen (see Fig 1). Within each condition the four targets could be discriminated from each other by a condition-specific features: (i) colour-alone (targets were a red-dot, blue-dot, yellow-dot and white-dot), (ii) shape-alone (black-cross, black-square, black-triangle and black-star) or (iii) shape-and-colour (blue-star, blue-triangle, red-star and red-triangle). This last condition is referred to as the ‘combined’ condition as it uses ‘combined-’ instead of ‘single-feature’ cues (i.e. shape AND colour need to be processed to differentiate between individual targets).

Right-handed participants began trials in these conditions by placing their stylus on the target in the bottom left hand corner. With a 2 second delay this triggered all targets to begin moving on the screen, following the same pseudo-random paths as in the single-target condition. At movement onset, participants were required to track the target in the bottom left corner with their stylus (i.e. the one they interacted with to commence the trial). Then for the remainder of the trial, whilst continuing to track, participants had to concurrently monitor cues that were programmed to appear in either the top or bottom left hand corners of the screen (see Fig 1 for example). In total two hundred cues per condition were presented (50 per trial) in a fixed pseudo-random order, each for 0.5 seconds with an inter-presentation gap of 1.8 seconds. Forty-eight of these cues were termed ‘valid’ (12 per trial) and were 80% sized versions of the targets within that condition. Participants were instructed that upon detecting a valid cue they were to shift their tracking behaviour immediately to the target matching the presented cue’s features, if they were not already tracking this target. The remaining 152 cues presented were ‘invalid’ black-dots. These interspersed the valid cues and were intended to encourage sustained vigilance to this concurrent sub-task. This use of interspersed invalid cues was methodologically analogous to the format of stimulus presentation used in traditional continuous performance tests (CPTs) of sustained vigilance (as in [17]). Valid cue presentations were counterbalanced for location (top or bottom corner), the duration of the gap between them (2 or 4 invalid cues between each valid) and instruction-type (e.g. bottom left to top left target). Left handed participants completed a mirrored version of the same task.

#### Data processing

The x- and y-coordinate position of the stylus on the screen (sampled at 120 Hz) during each trial were post-processed using custom-written scripts in MATLAB^™^ (version 7.10.0 R2010a, The MathWorks Inc). Within the cue-detection condition trials, the recorded time series was differentiated into two qualitatively different components: (i) ‘tracking periods’ in which participants’ movement indicated they were tracking one of the four on-screen moving targets and (ii) ‘switching periods’ in which they were reorienting their tracking from one target to another, typically in response to a cue presentation. These two behaviours were discriminated using an algorithm that analysed the frequency and duration of time the stylus spent moving between and residing within each of the four quadrants of the screen, each target’s motion being lawfully bounded within a separate quadrant. More detailed explanations of the parameters and programming underpinning this and all other post-processing algorithms are explained in S1 Text.

*Tracking outcomes*: Tracking performance within the single-target condition trials and the tracking periods of the cue-detection condition trials was initially described using two outcome measures, which were derived from a continuous measurement of ‘Tracking Error’: the straight-line distance between the centre of the target being tracked and the participant’s stylus (measured in millimetres). This error value was calculated for each sampled time-point, square-root transformed to adjust for positive skew and then for each trial the time-series of error values was summarised by its mean and standard deviation, hereafter these two values are termed as that trial’s (mean) Tracking Error (TE) and Intra-Individual Variability (IIV) scores, respectively. These two outcomes were then mean averaged across the four trials within a given condition. Subsequently it was observed that within all conditions TE and IIV were significantly correlated with one another (*r* = .312 to .512; *p* ≤ .03). Consequently, standard deviation values (IIV) were also linearly regressed against their corresponding means values (TE) and the resultant unstandardized residuals were used as a further measure of IIV, which quantified the amount of unexplained variability in a participant’s performance after controlling for variability that was related to the central tendency of a participant’s tracking ability. The metric is hereafter termed the Residual Intra-Individual Variability (RIIV). To summarise, higher scores on these outcomes indicated poorer average accuracy (TE) and greater fluctuations in accuracy across time; overall (IIV) and after partialling out shared variation with average accuracy (RIIV).

For each Cue Detection condition an additional three outcomes were generated by subtracting a participants’ TE, IIV and RIIV performances on the single-target condition from their corresponding scores on the cue-detection conditions. These difference scores, termed ‘ΔTE’, ‘ΔIIV’ and ‘ΔRIIV’ indicated the change in a participant’s TE, IIV and RIIV scores on a given cue-detection condition relative to the single-target condition. These were referred to as the ‘Cue-detection Cost’ scores in relation to these outcomes for a given condition.

*Cue-detection outcomes*: Data from switching periods within the cue-detection conditions’ trials was cross-referenced against the known time-points during these trials when valid cues were presented to generate the following three measures: (i) correct reactions, defined as the number of valid cue presentations that were correctly responded to; (ii) false reactions, the number of switches to targets in the absence of a valid cue; (iii) mean reaction time in seconds (calculated for correct reactions). As with the tracking outcomes, mean reaction time was averaged across the four trials within a given condition. Correct and false reactions were summed within condition and correct reactions were further converted into a percentage score (CR-Score), indicative of the proportion of the total number of valid cues presented within the condition that were correctly responded to.

During all reactions (correct or otherwise), movement of the stylus had to be consistent with moving from one target’s quadrant to another for a period of time too long in duration to plausibly reflect a chance ‘drift’ briefly out into a neighbouring quadrant whilst remaining on-task tracking the same target throughout (see S1 Text for details of how this definition was operationalised programmatically). Within some participants’ time-series there were occasional instances where it was not possible to confidently determine whether movement represented genuine switching or unintentional ‘drifting’ behaviour. These ‘Indeterminate Movements’ (IMs) were excluded from further analysis because they could not be classified with confidence as either tracking or switching. However, these were very rare occurrences and short in duration. Within each cue-detection condition the median number of IMs produced by a participant was 0 (IQR = 0 to 1). At most, participants were observed to produce 3 IMs within a given condition and even then these were a small proportion of the data produced. This is illustrated by the maximum percentage of data-points excluded due to IMs within any one participant’s data (by condition): colour = 2.1%; shape = 1.7%; combined = 1.2%.

#### Analysis

Preliminary data exploration was conducted using a standardised protocol [50] and confirmed TE, IIV, RIIV, ΔTE, ΔIIV, ΔRIIV, CR-Score and mean reaction time were all suitable for analysis as continuous dependent variables. The dataset for this experiment is provided in S1 Dataset and the raw data post-processed to produce these outcome measures is available on request from the corresponding author. The α level for all statistical analysis was set at p < .05 and performance on each of the dependant variables was analysed using multi-level linear modelling (MLM) techniques in SAS (version 9.4, SAS Institute, 2012).

For a given dependent variable, a hypothesis-derived initial model was first generated and subsequently refined through a backwards elimination process to remove factors/interactions that were not significantly related to performance. At each step of the backwards elimination the main effect or interaction with the largest non-significant *p*-value was removed from the model, unless it was a non-significant main effect involved in a significant two-way interaction or its removal resulted in a large reduction of the goodness of fit of the model (Akaike’s Information Criteria score rose by ≥ 10 [51]).

For the three MLMs that specified TE, IIV and RIIV, respectively, as their dependent variable a maximum likelihood method was used to estimate the initial model. Each specified both condition (single-target, colour, shape or combined) and counterbalance-order (1^st^, 2^nd^, 3^rd^ or 4^th^) as fixed repeated factors, nested within participants, as well as a 2-way interaction between these factors. The five MLMs respectively specifying ΔTE, ΔIIV, ΔRIIV, CR-Score and mean reaction time as their dependent variables followed the same specifications, with two minor modifications to adjust for these outcomes being measured exclusively within the three cue-detection conditions. Firstly, the condition factor within these MLMs was readjusted to have three rather than four levels (colour, shape or combined). Secondly, an additional between-subject fixed factor was included in the initial model that categorically described the number of false reactions participants made, along with a 2-way interaction between this factor and condition. In relation to false reactions: in this experiment quantitatively few were produced by participants, prompting conversion of this variable into a categorical format and analysis of it as a potential moderator of performance, rather than analysing it as a separate dependent variable. The average number of false reactions across conditions was categorised into three bands (≤ 0.5; > 0.5 but ≤ 1; > 1). This gave an approximately equal distribution of participants across these levels within the sample.

### Results

#### Tracking outcomes

Post backwards elimination, the mixed linear models analysing Tracking Error (TE), its total (IIV) and residual (RIIV) Intra-Individual Variability all retained significant main effects of condition (for TE: *F*(3,48) = 210.29; *p* < .001; for IIV: *F*(3,48) = 160.88; *p* < .001; for RIIV: *F*(3,48) = 42.23; *p* < .001) and, in the case of TE and RIIV, Counterbalance Order (for TE: *F*(3,48) = 3.92; *p* = .014; for RIIV: *F*(3,48) = 6.03; *p* = .001). Post-hoc pairwise comparisons (Tukey’s tests) were used to investigate further differences in performance during the single-target condition versus each of the cue-detection conditions on these outcomes. These consistently indicated significantly better performance for TE, IIV and RIIV when participants only had a single target to track (Table 1). Similar post-hoc analysis of the main effect of counterbalance found comparatively higher TE but lower RIIV in the first condition compared to some of the latter ones but this was not consistent across all comparisons or indicative of a linear trend (see S1 File).

**Table 1:** Post-hoc Tukey’s test pairwise comparisons of single-target versus cue-detection condition performance for tracking outcomes in Experiment 1. Legend: ‘TE’ = Tracking Error; ‘IIV’ = Intra-Individual Variability; ‘RIIV’ = Residual Intra-Individual Variability; ‘combi.’ = Combined Targets. Values in brackets are 95% Confidence Intervals.

	TE (mm^0.5^)		IIV (mm^0.5^)		RIIV (mm^0.5^)	
pairwise contrast	mean difference	*p*	mean difference	*p*	mean difference	*p*
colour—single	0.38 [0.34,0.43]	< .001	0.15 [0.12,0.17]	< .001	0.06 [0.04,0.08]	< .001
shape—single	0.39 [0.33,0.45]	< .001	0.18 [0.15,0.22]	< .001	0.09 [0.07,0.11]	< .001
combi.–single	0.53 [0.47,0.59]	< .001	0.20 [0.17,0.23]	< .001	0.08 [0.06,0.11]	< .001

A similar pattern of results was observed in the MLMs that analysed the relative size of the cost in TE, IIV and RIIV (ΔTE, ΔIIV and ΔRIIV) that each cue-detection condition elicited (i.e. tracking performance after controlling for single-target condition ability). Again, after backwards elimination the final models included significant main effects of condition (for ΔTE: *F*(2,48) = 62.33; *p* < .001; for ΔIIV: *F*(2,46) = 27.07; *p* < .001; for ΔRIIV: *F*(2,48) = 8.72; *p* < .001) and counterbalance order (for TE: *F*(3,48) = 3.25; *p* = .030; for ΔIIV: *F*(3,46) = 4.33; *p* = .009; for ΔRIIV: *F*(3,48) = 8.22; *p* < .001), whilst the model for ΔIIV also included a main effect for false reaction category, *F*(2,46) = 3.81; *p* = .030. Further exploration of the differences between cue conditions was conducted using Tukey’s test pairwise comparisons, which indicated consistently greater costs for ΔTE and ΔIIV in the combined, as opposed to either of the single-feature cue conditions (Table 2). Meanwhile, differences due to counterbalance order were consistent with worsening performance with time (i.e. a fatigue effect) only for ΔRIIV (see S1 File). The only statistically significant effect of false reaction (FR) category was that less ΔIIV cost was exhibited by those who average <0.5 FRs compared to those who average at least 1 FR per condition, (mean difference [95% CI] = 0.06 mm^0.5^ [0.01, 0.11], *p* = .023).

**Table 2:** Post-hoc Tukey’s test pairwise comparisons of differences in performance between cue-detection conditions for tracking and cue detection outcomes in Experiment 1.

	ΔTE (mm^0.5^)		ΔIIV (mm^0.5^)		ΔRIIV (mm^0.5^)		CR-Score (%)	
pairwise contrast	mean difference	*p*	mean difference	*p*	mean difference	*p*	mean difference	*p*
shape—colour	0.01 [-0.03,0.05]	.805	0.03 [0.01,0.05]	.001	0.03 [0.01,0.05]	< .001	6.39 [2.39,10.389]	.001
combi.–colour	0.15 [0.11,0.18]	< .001	0.02 [0.04,0.08]	< .001	0.02 [<0.01,0.04]	.012	-22.55 [-19.55,-25.56]	< .001
combi.–shape	0.14 [0.10,0.18]	< .001	0.06 [<0.01,0.05]	.040	-0.01 [-0.03,0.01]	.651	-28.93 [-25.80,-32.06]	< .001

‘ΔTE’ = cue-detection cost in Tracking Error; ‘ΔIIV’ = cue-detection cost in Intra-Individual Variability; ‘ΔRIIV’ = cue-detection cost in Residual Intra-Individual Variability; ‘CR-Score’ = percentage of valid cues correctly responded to; ‘combi.’ = combined targets. Values in brackets are 95% Confidence Intervals.

#### Cue-detection outcomes

**Fig 2:**
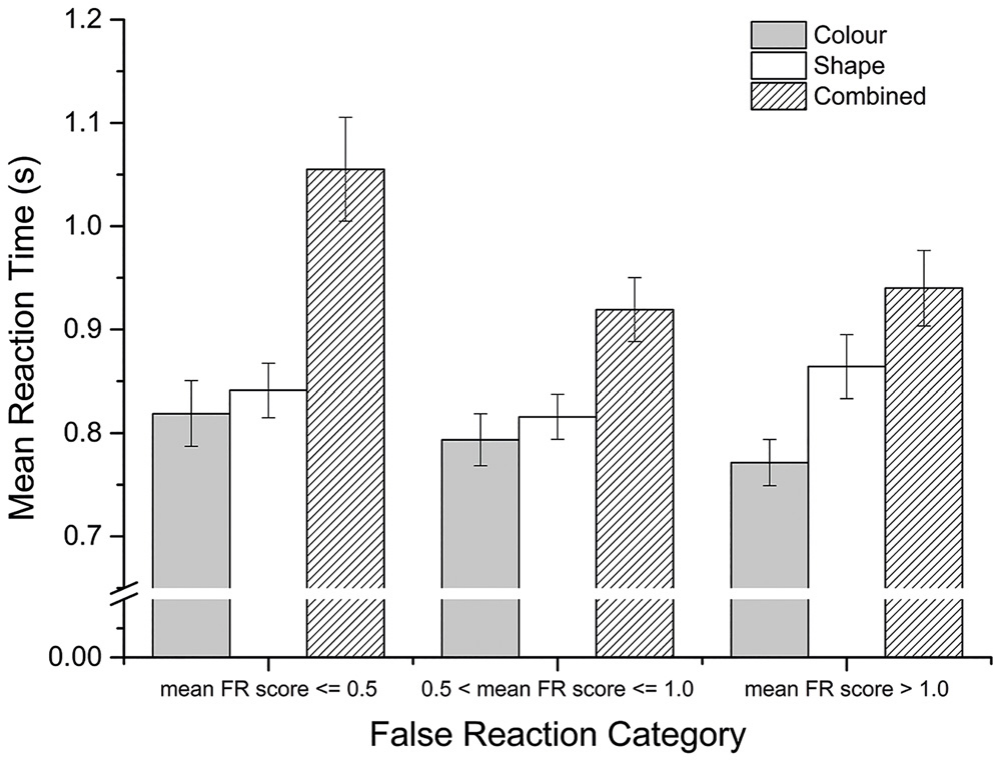
Bar graph depicting the significant interaction between condition and false reaction (FR) category for mean reaction time in Experiment 1. Error bars are 95% confidence intervals.

The MLM analysing the percentage of valid cues correctly reacted to (CR-score) as its dependent variable retained, post backwards elimination, only a main effect of condition, *F*(2,48) = 205.38; *p* < .001. Post-hoc pairwise comparisons of this main effect are reported in Table 2 and follow a similar pattern to that already described for ΔTE. The MLM that investigated mean reaction time also included a main effect of condition, *F*(2,46) = 60.67; *p* < .001, and retained a significant interaction between condition and false reaction category, *F*(4,46) = 6.09; *p* < .001. See Fig 2 for an illustration of this interaction.

### Discussion

The results indicated that performance on the VMA task was sensitive to experimental manipulations in adult participant’s attention load. When a cue-detection task was completed concurrently alongside manual tracking there were clear decrements in both the mean and variance of their tracking error (i.e. TE and IIV, which were both highly correlated with one another). There were also further effects on intra-individual performance variability that were not associated with mean tracking performance (i.e. residual IIV [RIIV]). These effects were irrespective of the specific cue- detection condition (Table 1), consistent with the principal of limited attentional capacity [46] and agree with previous research showing that performance on manual tracking task is detrimentally affected by placing competing demands on attentional resources [32–34].

In addition to these ‘gross’ manipulations of attention, evidence was also found for the salience of the specific visual cues within the cue-detection tasks influencing tracking performance. The size of the cue-detection costs in terms of mean tracking error (ΔTE) and its variability (ΔIIV) were qualitatively predicted by feature integration theory [47,48], with costs to performance levels on these outcomes significantly greater when tracking had to be performed alongside a ‘combined’ as opposed to ‘single-feature’ cue detection task. This systematic worsening of performance was also found in the percentage of valid cues responded to correctly (CR-score, see Table 2), suggesting performance on both tasks within the dual-task were similarly (negatively) affected by the increased attentional demands of combined-feature cues.

Importantly, these differences in tracking inaccuracy arose even though data-points that represented ‘actively responding’ to cues were excluded from this analysis. In other words, the differences occurred during phases of the dual-tasks when there was ‘passive’ visuo-cognitive rather than ‘active’ visuomotor competition being generated by the concurrent cue-detection task. Previous research has found that the deficits to manual coordination incurred by concurrent competitor tasks tend to be greatest when these tasks elicit additional motor responses (e.g. conversation interferes with manual coordination most during periods when a participant begins to speak and least around periods when they begin to listen [34]). Such ‘direct’ within-domain interference is unsurprising. What this experiment suggests is that further ‘indirect’ interference generated by competing visuo-cognitive demands is also possible, even when these demands do not require overt changes to visuomotor behaviour. Moreover, the extent of this interference appears to be associated with competing cognitive rather than visual demands (i.e. differences between cue-detection conditions arose due to variation in cue complexity). This finding suggests difficulty in disassociating cognitive and motor aspects of function entirely from one another and highlights the importance of assessing a person’s attentional functioning within the context of their goal-directed behaviour.

Further between condition effects were also found in participant’s residual intra-individual variability (ΔRIIV) but these were inconsistent with Feature Integration Theory. A significant difference in residual variability was only found between one of the single-feature conditions (colour but not shape) and the combined condition (Table 2). Additional differences in performance between the two single-feature cue conditions were observed during the study: whilst participants maintained equivalent levels of mean tracking performance in both single-feature conditions, they made a small but significantly greater proportion of correct responses to cues in the shape condition compared to the colour condition whilst inversely showing greater intra-individual variability on this condition (Table 2). This response pattern could plausibly be explained by participants using different monitoring strategies to switch attentional focus between the two competing tasks depending on whether cues were distinguished by shape or colour [52,53], thus demonstrating a level of detail in analysis that traditional standardised assessments of attention [13–16] are relatively ill-equipped to investigate.

Similarly, for mean reaction time the main effect of condition was complicated by an interaction with the average number of false reactions per condition, a measure likely to represent a participants’ inhibitory control [54,55]. This interaction suggested that the difference between the mean reaction time for combined- and single-feature cues was greater in participants who made fewer cue-detecting error. Again this may reflect different strategic approaches to managing the simultaneous demands of tracking and cue detection and suggests that cue-detection conditions, as intended, assessed the efficiency with which participants’ executively managed distributing their attentional resources. This is a highly plausible interpretation given that response inhibition and task switching [56] are both clear executive demands upon attention within the cue-detection conditions.

In summary, Experiment 1 establishes ‘proof of concept’ that outcomes recorded via this novel visuomotor methodology are meaningful indices of executive attentional functioning in adults. The potential for this more nuanced assessment of ‘visuomotor attention’ to enable deeper understanding of attention’s role in visuomotor behaviour is suggested by the facts that: (i) many existing standard assessment methodologies (e.g. [17]) are more limited in their abilities to analyse cognitive-motor interactions or differentiate between mean and intra-individual variability of performance as independent indices of attentive functioning; (ii) the specific type of behaviour we examined (manual tracking) has historically been defined purely as a non-attentional ‘motor’ skill [41–43].

## Experiment 2

### Introduction

Experiment 2 sought to adapt the method described in Experiment 1 into a format suitable for use as a standardised psychometric assessment within community-based settings and evaluate its construct validity as an assessment of visuomotor attention in adults and children. Specific adaptations were as follows: cue saliency was increased to reduce task difficulty; administration time was reduced by only presenting one of the three original cue-detection conditions (shape cues) and a fixed, rather than counterbalanced, order was imposed, with single-target tracking performance measured in two blocks either side of the dual-task cue-detection condition.

This revised method was trialled to determine whether it retained the ability to detect principled manipulations in the attentional load of adults (i.e. single-target versus cue-detection conditions) and was also capable of detecting similar effects in children. The method’s ability to detect differences in performance between these two age groups was also investigated. Substantial evidence suggests that children’s gross motor performance is more affected than adults by dual-task manipulations [57–59] and that executive attention abilities do not reach full maturity until late adolescence [60]. Consequently, it was predicted that age differences in manual tracking should be selectively greater within the cue-detection condition and that children would perform least well compared to adults on the cue-detection condition; providing further evidence of the assessment’s specific ability to detect principled changes in visuomotor attention.

### Methods

#### Participants

Two groups of participants were recruited for this study. The first was an opportunity sample of 53 self-reported healthy University of Leeds undergraduate students, recruited through informal networks (36 male, 17 female; median age [range] = 21 [19 to 26]). The second was a cohort of 47 children recruited through two after-school/holiday play-schemes based in Aberdeen, North East Scotland. Children and parents/guardians within this cohort gave permission for data to be collected from them for use in this Experiment and the two subsequent experiments described in the paper (i.e. Experiments 3 and 4). In relation to this experiment only a subsample of 32 children from the larger cohort (17 male, 15 female; median age [range] = 9 [7 to 12]), who were classified as ‘Typically Developing’ children, were included in the analysis. Typical development was judged using a standardised child mental health questionnaire completed by parents at the point of consent (The Strengths and Difficulties Questionnaire [SDQ]) [61] and defined by a score of 16 or less on the SDQ’s Total Difficulties Score scale [62] and a response of ‘no’ to following question on the SDQ supplementary Impact statement: ‘Overall, do you think your child has difficulties in one of the following areas: emotional, concentration, behaviour and being able to get along with other people?’ [63]. In this and both the two subsequent experiments reported in this paper: all participants had normal or corrected to normal vision; volunteered without incentive offered and gave informed written consent, as did parents/guardians in instances if participants were children.

#### Materials and procedure

Whilst seated comfortably at a desk of appropriate height for their age, participants completed a shortened version (15–20 min) of the VMA task described in Experiment 1. For all participants the same fixed order of presentation was used: a single-target tracking trial (ST1), four cue-detection (CD) trials and another single-target trial (ST2). All trials were 2 minutes long in duration. The ST1 trial was identical to the first trial from the ‘single-target’ condition in Experiment 1, whilst ST2 was a copy of the second. The four intervening CD trials were identical to those described as the ‘shape-target’ cue-detection condition in Experiment 1, except cue stimuli were increased in size to 120% the size of the moving on-screen targets, increasing their salience. Prior to participating, all participants watched a set of pre-recorded instructions on the tablet (3 min) and performed two brief (30 seconds per condition) practice trials under supervision to confirm comprehension of instructions.

#### Data processing

Participant’s raw output on each trial was post-processed to calculate their performance on the same outcome measures as described in Experiment 1. That is, participant’s manual tracking error during each trial was measured in terms of its mean (termed: tracking error [TE]) and standard deviation (intra-individual variability [IIV]), as well as the residual intra-individual variability after partialling out shared variance with TE [RIIV]. For cue detection skills, the number of correct reactions to valid cue presentations; their mean reaction time during these responses and a categorical (Yes/No) assessment of whether they made any false reactions during response were recorded.

Each participant produced six measures of TE and six of RIIV measures: one per metric for each of the two single-target trials and one per metric for each of the four cue- detection (CD) trials. They also had four measures of their cue detection outcomes: one each per CD trial. As in Experiment 1, performance on TE, IIV, RIIV and mean reaction time outcomes across all four CD condition trials was collapsed into a mean average for each outcome. Meanwhile, the number of cues correctly reacted to during these trials was summed and presented as a percentage ‘CR-score’. Preliminary investigation of performance on the first and second Single Target Trials (ST1 and ST2) trials indicated equivalent ‘condition averaging’ of TE, IIV and RIIV outcomes across these two trials would not be appropriate (Table 3). This was because MLM analyses indicated that there were significant differences in performances on all three tracking outcomes from ST1 to ST2 (TE: *F*(1,83) = 37.36; *p* < .001; IIV: *F*(1,83) = 22.80; *p* < .001; RIIV: *F*(1,83) = 7.27; *p* = .009). Therefore, tracking performance on the VMA Task was differentiated into three ‘stages’: The first single-target trial (ST1), the following cue-detection condition in its entirety (CD) and the final single-target trial (ST2).

**Table 3:** Summary of performance on tracking and cue-detection outcomes for Experiment 2.

		1st single-target trial		Cue-detection condition		2nd single-target trial	
Outcome measures		Adults	Children	Adults	Children	Adults	Children
Tracking	TE (mm^0.5^)	3.01 (0.44)	3.79 (0.34)	3.26 (0.48)	4.39 (0.37)	3.13 (0.50)	3.98 (0.40)
		*2*.*97 (0*.*37)*	*3*.*77 (0*.*30)*	*3*.*20 (0*.*38)*	*4*.*36 (0*.*32)*	*3*.*07 (0*.*43)*	*3*.*94 (0*.*37)*
	ΔTE (mm^0.5^)			0.25 (0.22)	0.61 (0.15)		
				*0*.*23 (0*.*21)*	*0*.*61 (0*.*16)*		
	IIV (mm^0.5^)	0.70 (0.09)	0.90 (0.11)	0.87 (0.12)	1.12 (0.17)	0.75 (0.14)	0.98 (0.18)
		*0*.*69 (0*.*08)*	*0*.*90 (0*.*11)*	*0*.*84 (0*.*07)*	*1*.*11 (0*.*17)*	*0*.*71 (0*.*08)*	*0*.*98(0*.*19)*
	ΔIIV (mm^0.5^)			0.17 (0.11)	0.21 (0.10)		
				*0*.*15 (0*.*08)*	*0*.*20 (0*.*09)*		
	RIIV (mm^0.5^)	-0.06 (0.13)	-0.02 (0.10)	0.05 (0.11)	0.07 (0.12)	-0.04 (0.15)	0.02 (0.14)
		*-0*.*05 (0*.*12)*	*-0*.*01 (0*.*10)*	*0*.*05 (0*.*09)*	*0*.*07 (0*.*13)*	*-0*.*05 (0*.*13)*	*0*.*03 (0*.*14)*
	ΔRIIV (mm^0.5^)			0.12 (0.09)	0.09 (0.09)		
				*0*.*13 (0*.*15)*	*0*.*04 (0*.*14)*		
Cue-detection	CR-Score (%)			72.6 (21.1)	56.8 (19.2)		
				*76*.*0 (17*.*8)*	*57*.*1 (19*.*4)*		
	mRT (sec)			0.58 (0.23)	0.92 (0.12)		
				*0*.*58 (0*.*23)*	*0*.*91 (0*.*11)*		

‘TE’ = Tracking Error; ‘IIV’ = Intra-Individual Variability; ‘RIIV’ = Residual Intra-Individual Variability; ‘ΔTE’ = cue detection cost for Tracking Error; ‘ΔIIV’ = cue detection cost for Intra-Individual Variability; ‘ΔRIIV’ = cue detection cost for Residual Intra-Individual Variability; ‘CR-Score’ = percentage of valid cues correctly responded to; ‘mRT’ = mean reaction time to valid cues. Note: values are means with standard deviation in brackets, the second row for each metric (in italics) is excluding outliers.

Cue-detection cost scores reflecting how much TE, IIV and RIIV performance were perturbed by dual tasking (ΔTE, ΔIIV and ΔRIIV) were again calculated. This time, the cost function was defined as the difference between the average measure during the CD condition and ST1. ST1 was selected as the more appropriate of the two single-target trials to use in this calculation because this typically represented participant’s best tracking performance (see Table 3), presumably because it was not confounded by fatigue. This dataset is available in full in S2 Dataset.

#### Analysis

TE, IIV and RIIV were each investigated separately as dependent variables using three MLMs. These models examined the following factors as fixed effects: age-group (adult or child) and stage (ST1, CD or ST2). An interaction between age-group and stage was also considered. The three measures of the amount of cost to tracking performance wrought by concurrent cue detection (ΔTE, ΔIIV and ΔRIIV) were also each analysed as dependent variables separately, using three between-subjects General Linear Models (GLMs). These GLMs specified age-group and false reactions (‘None’ versus ‘≥ 1 False Reaction’) as independent categorical factors, with a 2-way interaction also explored. Finally, two further equivalent GLMs were generated to analyse CR-Score and mean reaction time, respectively, as outcomes.

### Results

Data exploration using boxplots and Cleveland dotplots identified four participants in the adult sample and one in the child sample as outliers on at least one of the trials for either their TE and/or RIIV responses. Their exclusion did not substantially alter the mean performance but did influence within age-group variability for some conditions (see Table 3), resulting in violations of the assumption of homogeneity of variance. Therefore statistical analysis was conducted with these cases excluded.

#### Tracking error

The MLM investigating mean tracking error (TE) reported a significant main effect of stage, *F*(2,78) = 178.63; *p* < .001, and age-group, *F*(1,78) = 139.02; *p* < .001, and a significant interaction between these factors, *F*(2,78) = 36.96; *p* < .001. Post-hoc pairwise comparisons (Table 4) indicated that the pattern of significant differences due to stage for TE, within both age-groups, was consistent with results from Experiment 1 (i.e. poorer tracking during the cue-detection than during the single-target trials).

**Table 4:** Post-hoc Tukey’s test pairwise comparisons for tracking error (TE) between single-target and cue-detection conditions in Experiment 2 split by age-group.

	Adults		Children	
pairwise contrast	mean difference	*p*	mean difference	*p*
CD—ST1	0.23 [0.15,0.31]	< .001	0.59 [0.49,0.69]	< .001
CD—ST2	0.13 [0.05,0.21]	< .001	0.41 [0.31,0.51]	< .001
ST2 –ST1	0.10 [0.02,0.19]	.005	0.18 [0.08,0.28]	< .001

‘ST1’ = 1^st^ Single-Target trial; ‘ST2’ = 2^nd^ Single-Target trial; ‘CD’ = Cue-Detection condition. Values in brackets represent 95% Confidence Intervals.

**Fig 3:**
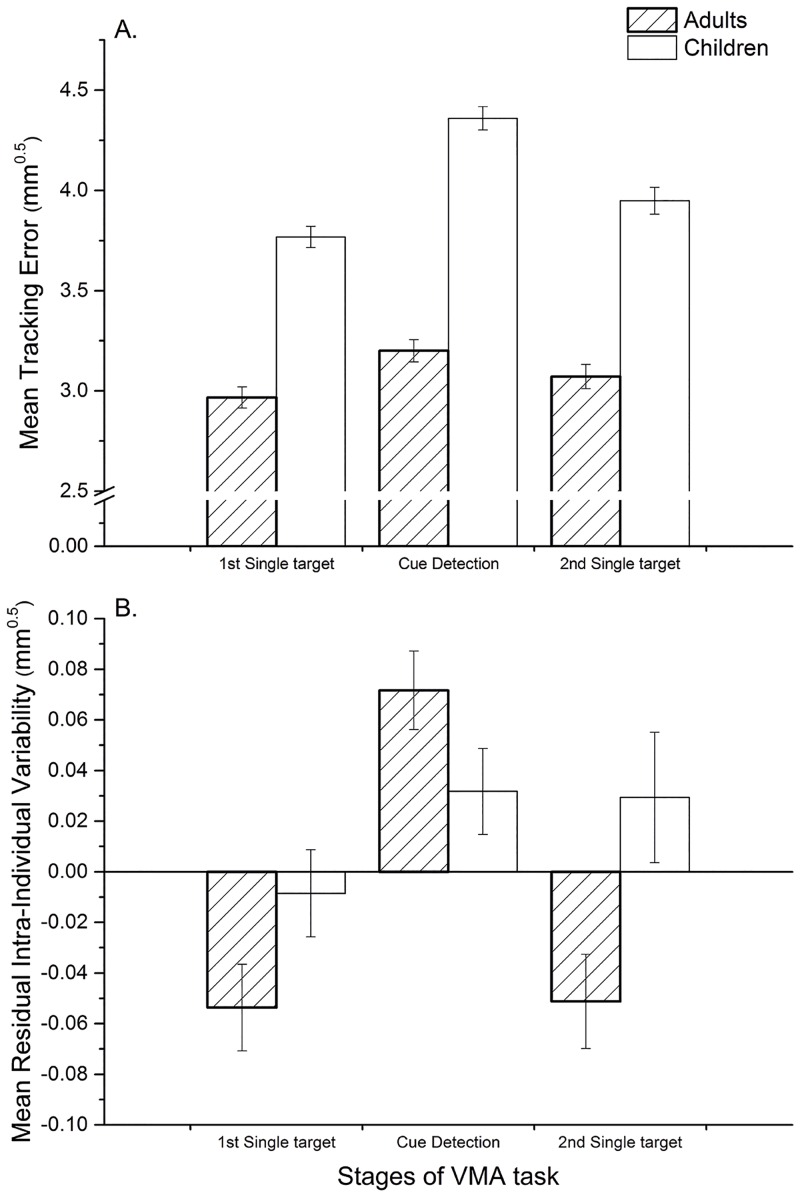
Bar graphs illustrating the significant interaction between age-group and VMA task stage for tracking outcomes in Experiment 2. Panel A: mean tracking error and Panel B: residual intra-individual variability. Error bars represent 95% confidence intervals.

Meanwhile, Fig 3 (Panel A) suggested the significant interaction was the consequence of more pronounced impairment in performance whilst dual-tasking in children compared to adults. This was investigated further by examining age differences in cue-detection cost for TE (i.e. ΔTE), with GLM analysis of this outcome reporting a significant main effect for age-group, *F*(1,76) = 71.26; *p* < .001; η_p_^2^ = .48, but no statistically significant effect of false reaction category (*p* = .051) or an interaction between these factors (*p* = .631). Inspection of age-group means and standard deviations for ΔTE (Table 3) indicated that performance deteriorated less as a consequence of concurrently performing cue detection in adults compared to children.

#### Intra-individual variability

For the measures of overall intra-individual variability (IIV and ΔIIV) the pattern of results approximated those already reported in relation to the mean tracking error (i.e. the effect of the experimental manipulations was comparable across all these outcomes). Therefore, for expediency, a detailed report of these results is given in S1 File.

For residual intra-individual variability (RIIV), the initial MLM results were similar to those already reported for tracking error: a main effect of stage, *F*(2,78) = 63.87; *p* < .001, and age-group, *F*(1,78) = 4.01; *p* .049, and a significant interaction, *F*(2,78) = 3.88; *p* = .025). For adults, post-hoc pairwise comparisons indicated that performance was significantly worse during the cue-detection condition relative to either the first (mean difference [95% CI] = 0.10 mm^0.5^ [0.07, 0.13], *p* < .001) or the second single-target tracking trial (mean difference [95% CI] = 0.10 mm^0.5^ [0.06, 0.14], *p* < .001). However, there was no difference in performance between these two single conditions (*p* = .850) and for children the only significant pairwise comparison indicated RIIV was significantly worse on the cue-detection condition compared to the first single-target trial (mean difference [95% CI] = 0.08 mm^0.5^ [0.04, 0.11]).

Fig 3 (Panel B) illustrates this interaction, which was explored further via analysis of the cue-detection cost function for this outcome (ΔRIIV). This GLM analysis found a significant main effect for age-group, *F*(1,76) = 6.56; *p* = .012; η_p_^2^ = .08 but no further effect of false reaction category (*p* = .900) or a significant interaction between these factors (*p* = .062). Estimated marginal means indicated that ΔRIIV score was significantly larger for adults than children (mean difference [95% CI] = 0.08 mm^0.5^ [0.02, 0.15]).

#### Cue detection

**Fig 4:**
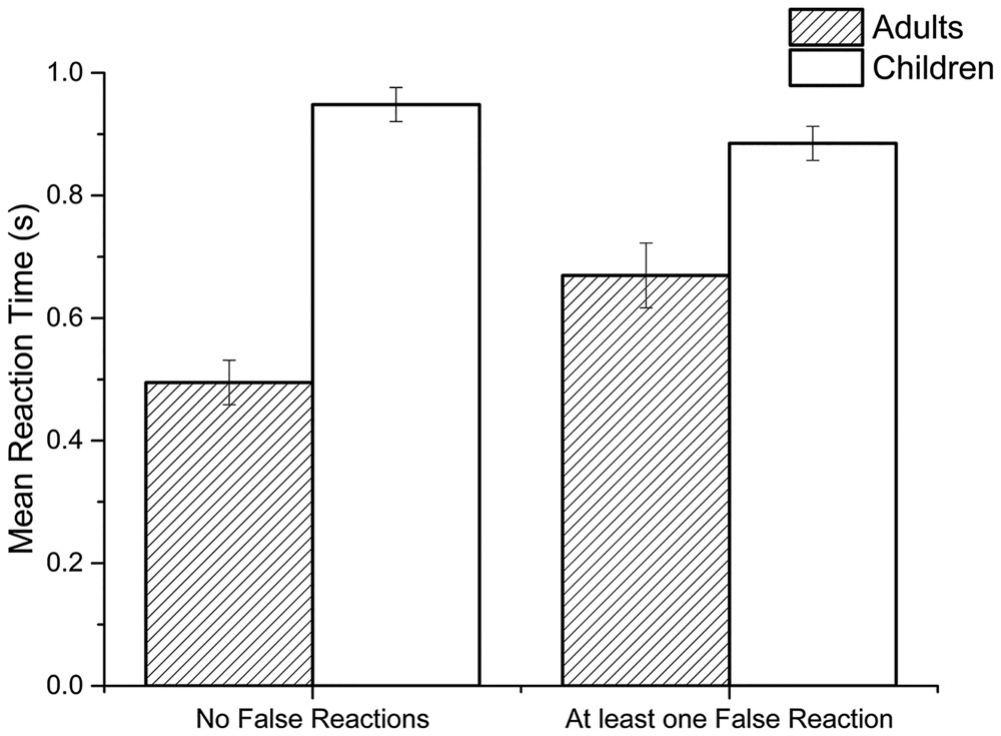
Bar graph illustrating the significant interaction between age-group and false reaction category for mean reaction time in Experiment 2. Error bars represent 95% confidence intervals.

Further GLM analyses of cue-detection condition outcomes found a significant main effect for age-group, *F*(1,76) = 19.90; *p* < .001; η_p_^2^ = .21, on the percentage of cues correctly responded too (CR-Score). Estimated marginal means indicating adults correctly responded to a significantly higher proportion of cues (mean difference [95% CI] = 19.1% [10.6, 27.6]). No further effects of false reaction category (*p* = .676) or an interaction between these factors (*p* = .488) were observed on this outcome. Meanwhile, for mean reaction time to cues a GLM analysis indicated a significant main effect of age-group, *F*(1,76) = 61.04; *p* < .001; η_p_^2^ = .45, interacting with the number of false reactions participant’s made (i.e. none vs at least one), *F*(1,76) = 7.72; *p* = .007; η_p_^2^ = .09, an interaction presented in Fig 4.

### Discussion

Consistent with Experiment 1, the mean error for manual tracking (Tracking Error [TE]) and its overall intra-individual variability (IIV) were always significantly greater during cue-detection relative to single-target tracking trials-suggesting this revised methodology was still sensitive to dual-task manipulations of attentional load. The results supported the prediction that age would modulate the size of the costs elicited by performing cue detection alongside tracking. Tracking performance improved with age over both conditions but significantly more so with respect to the cue-detection condition, illustrated by the higher ‘spike’ in tracking error generated by children during the cue-detection condition (Fig 3, Panel A). This finding is further supported by the significantly larger cue-detection cost in this (ΔTE) outcome within children and a greater proportion of cues being responded to correctly by adults than children.

In adults, the effect of dual-tasking on residual IIV also showed this ‘spike’ pattern. In children though, this proportion of their overall variability was typically higher when tracking single-targets and appeared to increase to a lesser extent when moving from the single-target tracking to the cue-detection condition but remained at this elevated level when moving back to single-target tracking at the end of the assessment (see Fig 3, Panel B). This possibly indicates more rapid fatiguing of the children’s capacity to sustain VMA. Overall, this response pattern suggests that the extent to which IIV is associated with mean tracking performance depends on whether attention is required to be sustained or divided and is influenced by age.

In adults, residual variability appeared to be minimal when participants were asked to focus purely on their tracking performance (i.e. single-target trials), with a principled increase in this component of variability coinciding with other factors being introduced into the task (i.e. concurrent cue detection). This suggest this component of IIV is a more specific indicator of executive rather than sustained attentional processes. In children, two key differences are noticeable: firstly, a greater proportion of their IIV appears to be unrelated to their mean tracking ability when single-target tracking, perhaps suggestive of children needing to exert more effortful executive control over their visuomotor behaviour than adults. This is consistent with evidence that the relative automaticity of motor skills does not peak until early adulthood [64]. Secondly, residual IIV seems to vary to a lesser extent over the three stages of the task. This may reflect children having more limited attentional resources available for redistribution (e.g. when moving from single-target to cue-detection conditions) and/or less strategic, executive control over how they redistribute their attention (e.g. when moving from cue-detection back to single target tracking). This proposal accords with previous research showing that aspects of executive control (e.g. set-shifting ability, inhibition control and cognitive flexibility) mature throughout childhood [60,65].

Consistent with Experiment 1, the results also indicated that reaction time to cues was moderated by the number of errors of commission (i.e. false reactions) participants made. Fig 4 illustrates that although adults were generally faster to react, the deficit between age-groups was smaller for participants who made errors in response compared to those who did not. These results suggest that higher levels of inhibitory control (i.e. no false reactions) came at greater costs to children than adults (i.e. slower reaction times)—again consistent with prior evidence of inhibition being an executive function that is less developed in children and therefore possibly more attentionally demanding [60,65].

In summary, Experiment 2 demonstrated the feasibility of adopting a more detailed and contextually specific assessment of sustained and executive visuomotor attention that, critically, can be presented in a standardised format for use with children in community-based settings.

## Experiment 3

### Introduction

A key difference between the approach developed in Experiments 1 and 2 and traditional assessment methods [26, 35–37] is that this novel methodology has been purposefully designed to evaluate how attentional processes operate in the context of guiding visuomotor action [66]. This context-specificity consequently prompted a third experiment, which investigates the methodologies’ capacity to explore interactions between sustained and executive attention and an individual’s visuomotor abilities. To address this question a group of children were recruited via an occupational therapy clinic for children with movement problems. These children were diagnosed with a variety of developmental disorders [67–70], all of which had contributed to these children having significant coordination difficulties (to the extent that they were referred to the clinic). This group’s performance on the VMA task was contrasted with that of a group typically-developing children to determine if they were more likely to make use of a ‘Loss-Based Selection’ strategy [71] whilst performing the task; a strategic framework previously developed to explain complex patterns of cognitive-motor interaction within dual-tasking.

Loss-Based Selection (LBS) is a specific model of cognitive resource distribution that explains the behaviour of individuals with sub-optimal motor control (e.g. children and the elderly) on dual-tasks that involve an element of gross-motor performance [72,73]. It stems from observations that such individuals often show less performance decrements on motor components of these dual-task and more impairment on concurrent cognitive components, whilst healthy adults exhibit more equitably distributed impairment across all components. The explanation proffered for these group differences is that children and the elderly are less equitable in their underlying attentional resource distribution during dual-tasks, prioritising maintenance of gross-motor performance over other task demands, because they can less afford further deterioration their already inferior visuomotor skills. This experiment’s hypothesis consequently predicts that the more an individual is taxed by the visuomotor coordination demands of a complex task the more likely they are to resort to such a strategy: biasing attention towards protecting their more vulnerable motor abilities, irrespective of instructions to balance cognitive effort equally across all task demands [72].

We speculated that participants with coordination difficulties would strategically choose to distribute their attention in a way that mitigates their compromised visuomotor capabilities, consistent with previous evidence in children and older adults showing that both groups prioritising gross-motor over cognitive control under dual-task conditions [72,73]. If this response pattern was also observed to be more prevalent in a clinical group compared to typically-developing children on this novel task, it would support the importance of viewing higher-level cognitive function and underlying sensorimotor control as factors that interact in a bi-directional fashion. It would also highlight the value of context-specific assessments such as this task that are uniquely suited to developing our understanding of such relationships.

### Methods

#### Participants

A group of 11 children (all male; 2 left handed; median age [range] = 9 years [7 to 13]) were recruited from an occupational therapy rehabilitation clinic for children with movement disorders, run in North Yorkshire. All had at least one clinically diagnosed developmental disorder resulting in overt movement problems and no co-occurring musculoskeletal impairments (e.g. cerebral palsy). Criteria for referral to the clinic was that these movement problems had a clinically significant impact on these children’s daily functioning. This ‘Coordination Difficulties’ group was contrasted with a ‘Typically Developing’ group that comprised of 11 participants selected from the group of 32 typically developing children recruited for Experiment 2, who matched the Coordination Difficulties groups as closely as possible for age, sex and handedness (all male; 2 left handed; median age [range] = 9 years [7 to 11]). This dataset is available in S2 Dataset).

#### Materials and procedure

Children in both groups participated by completing the VMA task whilst attending their occupational therapy clinic and after school club, respectively. Materials and procedure for the task were as described in Experiment 2, with the same outcome measures for tracking and cue detection calculated. Note: for the typically developing participants this constituted further analyses of the datasets already collected from them.

#### Analysis

The primary tracking outcomes (mean tracking error (TE), its intra-individual variability (IIV) and residual intra-individual variability (RIIV)) were each investigated separately as dependent variables using Mixed Linear Modelling (MLM). MLMs were fully factorial and specified the following factors as fixed effects: group (coordination difficulties or typically developing) and stage (1^st^ single-target trial [ST1], cue-detection condition [CD] and 2^nd^ single-target trial [ST2]).

Performance during the cue-detection stage of the VMA task was further investigated in two ways. Firstly, cue-detection costs to tracking performance (ΔTE and ΔIIV and ΔRIIV) were analysed as dependent variables using independent-samples Mann-Whitney U tests, with group specified as the between-subjects factor. Secondly, cue detection during the cue-detection stage was evaluated using three further Mann-Whitney U tests (also specifying group as the between-subject factor). These designated percentage of correction reactions (CR-score), mean reaction time and number of false reactions as their respective dependent variables. In contrast to Experiment 2, these more conservative (non-parametric) analytical tests were used to mitigate for the increased degree of in-group response variability on the task and the comparatively small sample compared to the previous experiment. Similarly, due to the small sample size it was also not feasible to handle false reactions as a potential categorical moderator variable. Few within this sample (3 out of 22) were classified as having made no false reaction errors. The number of false reactions was therefore instead analysed as an additional outcome variable.

### Results

Data exploration using boxplots and Cleveland dotplots identified 3 clear outliers in the coordination difficulties group and one in the typically developing outliers in terms of TE, IIV and/or RIIV responses. Given exclusion of these participants did not alter the significance of results but did compromise the matched nature of the sample and reduced the already limited number of observations, results are reported with these individuals retained in the analyses. On a related point, it was noticeable that the coordination difficulties group was markedly more heterogeneous in terms of manual-tracking performance, irrespective of whether outliers were excluded (see descriptive statistics in Table 5).

**Table 5:** Summary of performance on tracking and cue-detection outcomes for Experiment 3.

		1st single-target trial		cue-detection condition		2nd single-target trial	
Outcome measures		Coordination Difficulties	Typically Developing	Coordination Difficulties	Typically Developing	Coordination Difficulties	Typically Developing
Tracking	TE (mm^0.5^)	4.34 (0.84)	3.79 (0.39)	4.48 (0.79)	4.41 (0.37)	4.89 (1.15)	3.98 (0.42)
	ΔTE (mm^0.5^)			0.14 (0.47)	0.62 (0.18)		
	IIV (mm^0.5^)	1.24 (0.51)	0.92 (0.12)	1.41 (0.44)	1.15 (0.19)	1.52 (0.76)	0.96 (0.14)
	ΔIIV (mm^0.5^)			0.18 (0.29)	0.23 (0.09)		
	RIIV (mm^0.5^)	0.03 (0.26)	-0.03 (0.18)	0.13 (0.30)	-0.10 (0.18)	0.04 (0.40)	-0.07 (0.18)
	ΔRIIV (mm^0.5^)			0.11 (0.18)	-0.08 (0.09)		
Cue-detection	CR-Score (%)			31.8 (20.1)	64.1 (18.8)		
	mRT (sec)			1.00 (0.16)	0.89 (0.09)		
	False Reactions			3.64 (2.69)	2.00 (1.55)		

‘TE’ = Tracking Error; ‘IIV’ = Intra-Individual Variability; ‘RIIV’ = Residual Intra-Individual Variability ‘ΔTE’ = cue-detection cost for Tracking Error; ‘ΔIIV’ = cue-detection cost for Intra-Individual Variability; ‘ΔRIIV’ = cue-detection cost for Residual Intra-Individual Variability; ‘CR-Score’ = percentage of valid cues correctly responded to; ‘mRT’ = mean reaction time to valid cues. Note: values are means with standard deviation in brackets.

**Fig 5:**
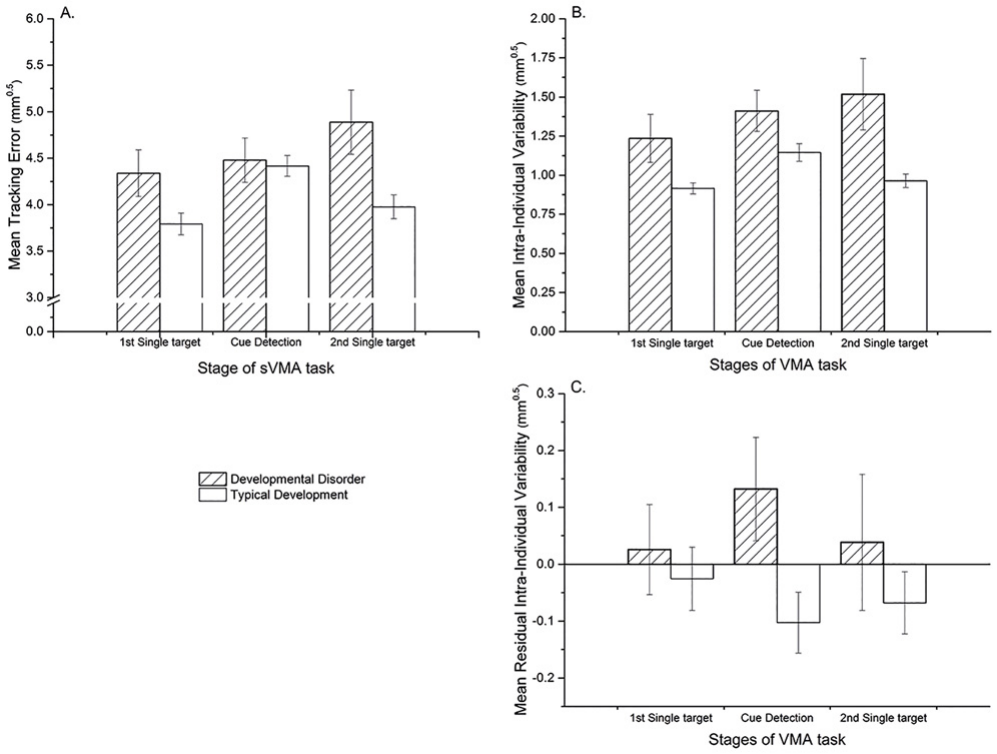
Bar graphs illustrating the significant interaction between group and VMA task stage for tracking outcomes in Experiment 2. Panel A: tracking error; Panel B: intra-individual variability; Panel C: residual intra-individual variability. Error bars represent 95% confidence intervals.

For tracking, MLM analysis of tracking error (TE), *F*(2,20) = 9.61; *p* = .001, overall intra-individual variability (IIV), *F*(2,20) = 3.88; *p* = .038, and residual intra-individual variability (RIIV), *F*(2,20) = 5.78; *p* = .010, all showed a significant interaction between stage and group. See Fig 5 (panels A, B and C) for depictions of these interactions. For TE, *F*(2,20) = 15.53; *p* < .001, and IIV, *F*(2,20) = 12.16; *p* < .001, but not RIIV (*p* = .632) a main effect of stage was also found; whilst only for IIV, *F*(1,20) = 5.76; *p* = .026, but not TE (*p* = .074) or RIIV (*p* = .204) a main effect of group was reported.

Mann-Whitney tests indicated that the cost in tracking error generated by performing cue detection concurrently (i.e. ΔTE) was significantly greater for the typically developing group than the coordination difficulties group (*U* = 104.00, z = -2.86, *p* = .003, *r* = 0.61). The percentage of correct reactions made was also significantly greater in the typically developing group (*U* = 105.00, z = 2.92, *p* = .002, *r* = 0.62). Further Mann-Whitney tests of the cue-detection cost outcomes indicated no significant differences between groups in overall intra-individual variability (*U* = 67.00, z = 0.43, *p* = .699, *r* = 0.09) but a significant difference in residual inter-individual variability (*U* = 18.00, z = -2.79, *p* = .005, *r* = 0.60). Meanwhile, the mean reaction time for responses to cues was not significantly different between groups (*U* = 32.50, z = -1.84, *p* = .065, *r* = 0.39), nor was the number of false reactions made (*U* = 38.50, z = -1.46, *p* = .151, *r* = 0.31). Means and standard deviations for each group on these outcomes are reported in Table 5.

### Discussion

In line with a priori predictions, the results suggest children with clinically recognised coordination difficulties exhibited a different pattern of response across the three stages of VMA task compared to typically developing controls. This is evident from the significant interactions between groups across the stages with respect to various aspects of their tracking performance (i.e. TE, IIV and RIIV). Visualisation of these interactions (see Fig 5, Panels A and B) indicate that the coordination difficulties group’s response pattern deviated from the typically developing group’s pattern and patterns previously reported in Experiments 1 and 2 for adults and children (e.g. see Fig 3).

Further analysis of outcomes within the cue-detection condition argue for the plausibility of these interactions arising from a ‘Loss-Based Selection’ response strategy [71] being adopted by the coordination difficulties group during this condition. We found an opposing direction in the group differences for cue-detection costs for mean tracking (ΔTE) and percentage correct reactions to valid cues (CR-Score). This contrasts with the consistent advantage that adults showed compared to typically developing children across both these outcomes in Experiment 2. Therefore in this study, rather than having a consistent advantage for minimising the impact of dual-tasking on mean tracking performance and cue detection rate, the coordination difficulties group show a selective advantage: minimising cost to tracking (ΔTE) but at the expense of detecting less cues (CR-Score) than controls. This pattern arose despite instructions to weight effort equally. The findings argue for greater recognition of the inherently cognitive demands of motor control [34,64], particularly in children with coordination difficulties, and suggest that compensatory attentional strategies are likely to arise in challenging situations where visuomotor resources are heavily taxed.

It is plausible that such compensatory strategies indicate an individual experiencing difficulty with executive management of competing motor and cognitive demands under dual-task conditions. This interpretation is consistent with the high co-occurrence of attentional problems amongst children with poor motor coordination [74,75] and the suggestion that difficulties in executive functioning is a key feature of both ADHD [76,77] and Developmental Coordination Disorder (DCD) [78]. This interpretation is further corroborated by the between group differences in IIV reported: overall response variability for visuomotor tracking is greater in the coordination difficulties group but in the dual tasking condition this deficiency appears increasingly to be attributable to residual IIV (i.e. variability not associated with mean tracking performance) compared to controls (See Fig 5 Panels B and C). This is noteworthy as it illustrates that a component of variability previously proposed as a measure of executive attention (Experiment 2) is selectively recruited to a greater extent in a group characterised by functional impairments in their visuomotor coordination.

There are, however, limitations to this study and the hypotheses proposed in this discussion will require further empirical investigation. Firstly, whilst all children in the clinical sample were uniform in having received a clinical referral to an occupational therapy clinic for significant developmental delay in their coordination skills, we were not able to gain access to these children’s confidential medical records to clarify the extent they also had further co-occurring developmental disorders. Co-occurrence within disorders of psychological development (a medical category that encompass disorders of motor and cognitive function) is common [79]. For example, children with DCD are more likely than not to meet the criteria, if assessed, for diagnosis of further developmental disorders [80]. Thus, it is highly probable that the aetiological causes of the coordination difficulties within our sample vary between individuals. Indeed, the broader response heterogeneity exhibited by children in the coordination difficulties group suggests that the strategic differences at an individual level should also be investigated systematically in future research. Lastly, we acknowledge that both groups were tested in surroundings that they were familiar with and attended regularly (e.g. their after school club or therapy clinic) but were non-standardised between groups, representing a potential source of bias.

This is, to our knowledge, the first account of Loss-Based Selection strategies affecting tasks involving fine-motor rather than gross-motor control [72,73], suggesting that the presence of such strategic processes may be a more generalised feature within motor planning than previously realised. This proposition appears reasonable in light of recent models developed for describing attention’s role in action [66,81] and is an observation that (again) undermines the validity of simplistic approaches taken to assessing cognition in many existing standardised test batteries [17,29,30].

## Experiment 4

### Introduction

In Experiment 4, the validity of the VMA task as an alternative to traditional standardised measures of attention is explored by relating VMA performance to performance on a set of existing assessments. Thus it tests earlier arguments for the construct validity of VMA as a meaningful standardised procedure for assessing a variety of attentional processes (Experiment 2); investigating evidence of its convergent validity with: (i) an established Continuous Performance Task (CPT) methodology and (ii) two parental-report questionnaires on inattentive and hyperactive behaviours, which that are extensively used in research and clinical practice to estimate children’s attentional capacity).

The most widely used methodology for assessing sustained attention has traditionally been the Continuous Performance Task (CPT) [17]. There are numerous versions of this type of psychometric test (see [17,18]) that are presented as “gold standard” measures of sustained attention and supported by large normative samples. CPT methodology asks participants to attend to a series of sequentially presented visual or auditory stimuli and to react to specific stimuli combinations within that series (e.g. a ‘1’ followed by a ‘9’ in a series of single digits). Performance is then summarised in terms of the total number of correct and incorrect responses made and participants’ mean latency of response. These measures of overall performance and central tendency of response are then treated as the primary indices of a participant’s capacity for sustained (continuous) attention.

However, as previously argued, intra-individual variability (IIV) is potentially a far more informative metric for representing a person’s capacity to *sustain* attention [19–24]. Responses to some CPTs can be used to measure IIV [19,82] but this is usually achieved through examining reaction time variability during a repetitive sequential presentation of trials. Using visuomotor manual tracking to measure sustained attention therefore represents an interesting methodological alternative because it captures moment-to-moment IIV whilst participants perform a genuinely continuous (rather than repetitive) behaviour [34].

Complementarily, the VMA task bears similarity to certain more-demanding CPT methodologies, such as the ‘Distractibility’ sub-test within the Gordon Diagnostic System [83]. This CPT modifies the classic ‘Vigilance’ CPT method by including additional ‘distractor’ stimuli presented intermittently on the screen throughout the task. Participants are instructed to ignore and inhibit responses to these distractors, thus making it more complicated and challenging. Consequently it is argued to be better able to detect individuals with only subtle deficits in sustained attention and inhibitory control [84]. The task demands for this CPT method are comparable to detecting cues within the VMA task in so far as: (1) both tasks require participants to pay attention to serial presentation of stimuli; (2) response to this series is only necessary under certain pre-specified conditions and (3) competing attentional demands undermine the ability to sustain attention on a single focal point. Thus it was expected that performance on comparable outcomes on these two tasks (e.g. number of responses to cues/stimuli, speed of these responses) should be contingent on similar underlying cognitive processes.

Performance on the VMA task was also compared against parental-report questionnaire evaluations of children’s attentive behaviours because these are routinely taken into account when clinically diagnosing attention disorders [85,86] and previous studies have concluded that these questionnaires demonstrate time- and cost-efficiency but lack objectivity when administered in isolation [8,9,87].

To summarise, this final experiment considered whether the VMA Task represents a valid alternative method for measuring pertinent aspects of childhood attention. To assess this, the strength of correlation between face-valid pairs of outcomes (e.g. reaction time to cues on both the VMA task and a CPT) was examined before exploratory principle component analysis (PCA) was conducted to evaluate the extent to which VMA Task performance was driven by the same set of latent variables as existing assessments. Lastly, the unique contribution that the VMA task could make to explaining variability in attentive behaviour (indexed by questionnaire response), over and above variability explained by more traditional assessment methods (indexed by CPTs), was explored using regression.

### Methods

#### Participants

The cohort of 47 children already referred to in Experiment 2 participated in this further study (27 male, 20 female; median age [range] = 9 years [7 to 12]). The only variation from that previous protocol was that all 47 child/parent responses were included for analysis in this study, because it did not apply the additional exclusion criteria relating to SDQ response described in Experiments 2 and 3. This full dataset is available in S2 Dataset.

#### Materials

Participants completed the VMA task exactly as described previously in Experiment 2, with the same tracking and cue-detection outcome measures calculated. The following additional assessments of attention were also completed:

*Continuous Performance Tests (CPTs)*: The two CPT-type subtests from the Gordon Diagnostic System (Dewitt, New York: Gordon Systems Inc.) were used (termed: ‘Vigilance’ and ‘Distractibility’). The Gordon Diagnostic System (GDS) is a standardised computerised test battery for assessing attention and inhibitory control [84] and performance on its CPT subtests have been normed in a sample of 1,266 four to sixteen year olds [83]. These measures correlate with other psychometric assessments of attention [88] and can be used to identify children diagnosed with ADHD with reasonable discriminant validity [89,90].

The GDS comprises of a plastic box (approximately 30 x 20 x 20 cm), housing a micro-processor, with an LED display screen and response button on the front. During its ‘Vigilance’ subtest participants attend to a pseudo-random sequence of single digits (1 to 9) displayed serially in the centre of the LED screen at a rate of one per second. Participants are instructed to react (by pressing the response button) as quickly as possible to presentations of the digit ‘9’ within this sequence, if the digit immediately preceding it in the sequence was ‘1’. In the ‘Distractibility’ subtest the same task is performed except the location that each digit presents on-screen is pseudo-randomly varied between one of three locations (centre, left or right). Participants are instructed to ignore digits presented to the left or right of centre and to continue to respond only to the digit ‘9’ providing it is (i) presented in the central position and (ii) the last digit prior to it that was presented centrally was a ‘1’ (irrespective of intervening peripheral presentations). Both subtests were 9 min in duration and each sequence included a total of 45 possible correct responses. Participants were explicitly instructed to try to avoid making errors in their response.

For each subtest the total number of correct reactions was recorded as well as the total number of commission errors (the number of times a participant pressed the response button in error). Raw scores for both total correct and commission errors were investigated as outcomes. Commission error scores were also age-normed and dichotomously categorised into either being ‘Typical’ or ‘Borderline/Abnormal’, based on suggested cut-offs in the Gordon Diagnostic System’s manual [84]. Mean response latency in seconds for each sub-test was also recorded.

*The Strengths and Difficulties Questionnaire (SDQ)*: The SDQ is a brief 25-item behavioural screening questionnaire for assessing child mental health [61]. Responses to its 5-item ‘hyperactivity’ subscale were used because large cohort studies in both the UK and Scandinavia [9,91] have shown that these have good sensitivity for identifying children with inattention and hyperactivity impairments (i.e. indicative of ‘combined’ subtype ADHD). This subscale’s brevity is noted to make it less effective at identifying individuals with difficulties in only one of these two domains [9].

*The Vanderbilt ADHD Diagnostic Parent Rating Scale (VADPRS)*: This rating scale specifically evaluates ADHD symptomology and its co-morbidities [85]. It has been endorsed for use within clinical practice by the American Academy of Paediatrics [92], after demonstrating acceptable levels of convergent validity with diagnostic clinical interviews for ADHD [85]. Parents in this experiment were asked to respond to items on the VADPRS’s Inattention and Hyperactivity/Impulsivity subscales (i.e. the two subscales which assess behaviours pertaining to the diagnostic criteria for ADHD). Responses to additional VADPRS subscales, which screen for commonly comorbid diagnoses (e.g. conduct disorder), were not collected to minimise the demands on parents’ time.

Participant’s scores were calculated for the Hyperactivity subscale (SDQ_Hyp._) of the SDQ (out of 10) and the separate Inattentive (VADPRS_Inat._) and Hyperactivity/Impulsivity (VADPRS_H&I_) subscales of the VADPRS (both out of 27). Higher scores on each of these scales implied more frequent expression of behaviours symptomatic of problems with attention. In instances where single item responses were missing this was substituted with the individuals’ median response value for the rest of that scale.

#### Procedures

The parental-report questionnaires were included within invitational packs that were distributed via the play-schemes to the parents of every eligible child. If parental consent was returned but without the fully completed questionnaires then at least two further attempts were made to request this additional information. Irrespective of whether complete questionnaire responses were obtained, all consenting children took part in two sessions that took place at approximately the same time of day (median discrepancy [IQR] = ± 46 [17–64] minutes) on different days. The VMA task was completed during one session and the GDS tasks in the other. Each session lasted 15–25 minutes and the order of them was counterbalanced between participants, stratifying for gender. Note: this reflects children undertaking a single assessment on the VMA-task that has then been analysed repeatedly in Experiments 2 to 4.

#### Analysis

Specific pairs of variables, which shared face validity, were identified from the VMA task and the Continuous Performance Tasks (CPTs). For pairings of continuous variables, the degree of association within a given pair was assessed using Spearman’s rank correlation coefficients, whilst Cohen’s Kappa was used to calculate the level of agreement within paired categorical variables.

Exploration of the latent factors explaining response variability was conducted using Principal Components Analysis (PCA) in SPSS (version 19.0.0, SPSS Inc.). Two PCAs were computed, the first explored common variance specifically within VMA task and Gordon Diagnostic System. It was planned to include all VMA task variables examined in the ‘paired’ analyses (i.e. CR-score, mean reaction time, false reactions, TE_ST1_, TE_ST2_) as well as the ‘cue-detection cost’ functions for the VMA task (i.e. ΔTE, ΔIIV and ΔRIIV) together with the following outcomes for both continuous performance tasks: total correct responses, commission errors (as continuous not categorical variables) and latency of response. Age (in years) was also included as a variable, to adjust for its likely additional influence on performance. A second PCA included the same VMA Task variables and age but this time in conjunction with the parental report questionnaire sub-scale scores (i.e. SDQ_Hyp._, VADPRS_Inat._ and VADPRS_H&I_). This analysis was completed separately from the first because 5 out of 47 participants lacked questionnaire responses; precluding them from inclusion in this second model. For both PCAs the number of factors extracted was decided after reviewing both (i) the scree-plot (identify points of inflection) and (ii) eigenvalues (at least > 1). Varimax rotation was applied to the factor loading to aid interpretation and within a given factor variable loadings were considered significant if >.40 [93].

Lastly, to explore further relationships identified in this second PCA three hierarchical linear regression models were created; each specifying one of the three questionnaire subscales as its dependent variable (i.e. SDQ_Hyp._, VADPRS_Inat._ and VADPRS_H&I_). As a first step in each of these models age and all outcome measures for the continuous performance tasks were entered, then at a second step the following two outcomes from the VMA task were entered: CR-Score and cue-detection cost for residual intra-individual variability (ΔRIIV). This second step evaluated whether these facets of VMA task performance explained unique variance in these questionnaire sub-scales after controlling for the predictors entered in the first step.

### Results

#### Pairwise comparisons

A significant correlation was observed in all but one of the eight pairings (Table 6). Five correlations fell within the ‘medium’ (i.e. .3 < *r* < .5) range in terms of their effect size, with the remaining two larger (*r* > .5) [93]. Cohen’s Kappa indicated significant agreement between the frequency of false reactions during the VMA task and age-standardised commission error classification for the Distractibility (*Ƙ* = 0.38; *p* = .005) but not Vigilance CPT (*Ƙ* = 0.07; *p* = .537). The statistically significant result indicates ‘fair’ agreement, which fell just short of ‘moderate’ agreement (0.40 to 0.60) based on Altman’s suggested benchmarks [94].

**Table 6:** Bivariate spearman correlations between pre-specified pairs of continuous variables from the VMA task and Continuous Performance Task (CPT) subtests.

		CPT Outcomes	
VMA Task Outcomes ^a^		Vigilance	Distractibility
		Total Correct	
Tracking Error (TE)	1^st^ single-target trial (TE_ST1_)	-.360*	-.520***
	2^nd^ single-target trial (TE_ST2_)	-.266	-.491***
Cue-detection	correct reaction score (CR-Score)	.398**	.458**
		Latency	
	mean reaction time	.464**	.627***

Values are Spearman Correlation Coefficients (*r*). ‘VMA’ = Visuomotor Attention; Bracketed terms denote abbreviated variable names.

^a^ n = 47.

*p < .05.

** *p* < .01.

***p < .001.

#### Principal Components Analysis (PCA)

For both PCAs the determinant of the correlation matrix was initially < 0.00001. On closer inspection this was identified as resulting from strong correlations between the measures of ‘cue-detection cost’ for mean Tracing Error and its intra-individual variability (ΔTE and ΔIIV; *r* = 0.906). To avoid problems with multi-collinearity [93] ΔIIV but not ΔTE was consequently excluded from these PCAs because sampling adequacy statistics indicated ΔTE’s retention was more beneficial to the validity of the model. Due to insufficient sampling adequacy it was also necessary to exclude ΔRIIV from the PCA investigating relationships with the continuous performance tasks (CPTs). After these exclusions both the first PCA (investigating the relationships between outcomes measures on the VMA task and CPTs [PCA1]) and the second (investigating relationships between outcomes measures on the VMA task and sub-scale scores on the SDQ and VADPRS questionnaires [PCA2]) had acceptable determinants for their correlation matrices (.003 and .005 respectively). The Kaiser-Meyer-Olkin (KMO) measure verified overall sampling adequacy in both PCAs (KMO_PCA1_ = 0.715; KMO_PCA2_ = 0.630) and for the individual items within each (KMO_PCA1_ > 0.555; KMO_PCA2_ > 0.501). These values are above the minimum acceptable value of 0.5 [93]. Bartlett’s test of spherecity was sufficiently large to justify using a PCA in both instances too (PCA1: χ^2^ (78) = 242.8, *p* < .001; PCA2: χ^2^ (55) = 195.7; *p* < .001).

For PCA1 both the scree-plot and eigenvalue concurred in suggesting a three component solution which cumulatively explained 60.2% of the total variation. The factor loadings for this PCA are reported in Table 7, which after rotation suggested the following relationships: The first factor related to performance on the total correct score of the ‘Distractibility’ variant of the continuous performance task (CPT) as well as the mean tracking error whilst tracking a single-target (both trials) and the cue-detection cost for this ability (i.e. ΔTE). Age also influenced this factor. The direction of weightings indicated that increased age was associated with better performance in terms of all these outcomes. On the second factor a positive relationship between measures of reaction time on both the VMA task and CPTs was observed, as well as a negative weightings for both the proportion of correct reactions made (CR-score) and ΔTE during the VMA task. This implied slower response speed associated with fewer correct responses but less cue-detection cost in tracking on the VMA task. Finally, the third factor primarily associated with errors in response on both tasks. Making no false reactions, rather than ≥ 1, on the VMA task was associated with making less errors of commission and more correct cue responses on both CPTs, as well as increasing age.

**Table 7:** Factor loadings for principal components analysis of VMA Task outcomes and Continuous Performance Task (CPT) outcomes (PCA1).

			Factor Loadings		
Variable			Factor 1	Factor 2	Factor 3
VMA Task Outcomes	TE_ST1_		**.83**	.33	-.15
	TE_ST2_		**.87**	.18	-.15
	ΔTE		**.50**	**-.45**	.30
	CR-Score		-.30	**-.50**	.35
	Mean reaction time		<-.01	**.73**	-.02
	False reactions		.39	-.28	**-.47**
CPT Outcomes	Vigilance subtest	Total Correct	-.18	-.22	**.56**
		Commission Errs.	.12	-.04	**-.79**
		Latency	.24	**.67**	.27
	Distractibility subtest	Total Correct	**-.49**	-.33	**.42**
		Commission Errs.	.05	-.18	**-.77**
		Latency	.24	**.86**	.07
Age			**-.68**	-.15	**.42**
Eigen values			2.80	2.60	2.43
% of total variance			21.5	20.0	18.7

Factor loading >.40 are in boldface and varimax rotation used. ‘VMA’ = Visual Motor Attention; ‘CPT’ = Continuous Performance Task; ‘TE_ST1_’ = Tracking Error during 1^st^ single-target trial; ‘TE_ST2_’ = Tracking Error during 2^nd^ single-target trial; ‘ΔTE’ = cue-detection cost for Tracking Error; ‘CR-Score’ = Percentage of valid cues correctly reacted to; ‘Commission Errs.’ = Commission Errors.

For PCA2, both the scree-plot and eigenvalue concurred in suggesting a three component solution which cumulatively explained 65.2% of the total variation. Table 8 reports the factor loadings for PCA2. The first factor was associated with age related change in VMA task performance, with younger children producing more tracking error on both single target trials (TE_ST1_ and TE_ST2_), detecting less cues (CR-Score) and being more likely to make false reactions. On the second factor there were consistent loadings for all the parental-report questionnaire subscales, which were negatively correlated with CR-score and cue-detection costs for residual intra-individual variability (ΔRIIV). That is higher (poorer) subscale scores associated with making fewer correct reactions but had smaller increases in a component of their tracking-task variability whilst the dual-tasking; this component being the portion of their overall intra-individual variability not associated with their mean tracking performance. Factor 3 showed loadings for all outcomes relating to the dual-task portion of the VMA-task. Making more correct reactions to cues was associated with faster reaction times but being more likely to make false reactions. These loadings also associated with having larger cue-detection costs for tracking error and smaller ones for residual intra-individual variability (ΔTE and ΔRIIV).

**Table 8:** Factor loadings for principal components analysis of VMA Task outcomes and standardised questionnaire responses (PCA2).

			Factor Loadings		
Variable			Factor 1	Factor 2	Factor 3
VMA task outcomes	TE_ST1_		**.89**	.11	-.08
	TE_ST2_		**.92**	.04	< .01
	ΔTE		.19	.10	**.49**
	ΔRIIV		.16	**-.54**	**-.52**
	CR-Score		**-.43**	**-.51**	**.50**
	Mean reaction time		.14	.10	**-.82**
	False reactions		**.43**	.22	**.45**
Questionnaire scales	SDQ	Hyperactivity	.01	**.83**	-.07
	VADPRS	Hyperactivity & Impulsivity	.10	**.90**	.06
		Inattention	.14	**.79**	.14
Age			**-.80**	<-.01	-.09
Eigen values			2.76	2.75	1.67
% of total variance			25.1	25.0	15.2

Factor loading >.40 are in boldface and varimax rotation used. ‘VMA’ = Visual Motor Attention; ‘TE_ST1_’ = Tracking Error during 1^st^ single-target trial; ‘TE_ST2_^’^ = Tracking Error during 2^nd^ single-target trial; ‘ΔTE’ = cue-detection cost for Tracking Error; ‘ΔRIIV’ = cue detection-cost for Residual Intra-Individual Variability; ‘CR-Score’ = Percentage of valid cues correctly reacted to; ‘SDQ’ = Strengths and Difficulties Questionnaire; ‘VADPRS’ = Vanderbilt ADHD Diagnostic Parent Rating Scale.

Subsequent exploratory analysis of cue-detection costs in residual intra-individual variability (ΔRIIV) revealed that performance on this outcome negatively correlated with the proportion of RIIV participants produced during their first single-target tracking trial (*r* = -.663, *p* < .001) but was not correlated with this same measure during the dual-task trials (*r* = -.105, *p* < .482). This indicates variation in cue-detection costs for this outcome were primarily a function of differences at baseline (i.e. their RIIV whilst single-target tracking).

#### Hierarchical linear regression

The results of the three hierarchical linear regression models, one per questionnaire subscale, are reported in Table 9. These analyses consistently indicated the variables entered at Step 1 (age and CPT outcomes) had no significant explanatory power for explaining response variability on any of the subscales. Meanwhile, Step 2 indicated significant increases in the proportion of variance explained after including additional predictors from the VMA-task, leading to an significant proportion of the variance overall being explained by these models in the case of the SDQ Hyperactivity subscale and VADPRS’s ‘Inattentive’ but not ‘Hyperactivity and Impulsivity’ subscale.

**Table 9:** Hierarchical linear regression reporting predictors of standardised questionnaire responses.

	SDQ: Hyperactivity subscale				VADPRS: Hyperactivity & Impulsivity subscale				VADPRS: Inattentiveness subscale			
	Step 1		Step 2		Step 1		Step 2		Step 1		Step 2	
Variable	*B* [SE]	β	*B* [SE]	β	*B* [SE]	β	*B* [SE]	β	*B* [SE]	β	*B* [SE]	β
Constant	4.72 [6.21]		9.57 [5.67]		9.48 [14.61]		20.50 [13.83]		-6.80 [14.35]		6.64 [12.57]	
Age	0.14 [0.31]	.09	0.03 [0.27]	.02	-0.40 [0.71]	-.11	-0.66 [0.65]	-.18	0.46 [0.70]	.12	0.13 [0.59]	.04
Tot. Correct_VIG_	-0.05 [0.10]	-.10	-0.05 [0.09]	-.09	0.02 [0.24]	.02	0.01 [0.22]	.01	-0.11 [0.23]	-.09	-0.12 [0.20]	-.10
Com. Errs._VIG_	0.06 [0.08]	.16	-0.04 [0.08]	-.12	0.33 [0.20]	.42	0.13 [0.20]	.16	0.08 [0.20]	.10	-0.15 [0.18]	-.18
Latency_VIG_	-0.90 [6.75]	-.03	-1.48 [6.04]	-.05	0.45 [15.72]	.01	-2.58 [14.51]	-.04	2.46 [15.43]	.04	-2.80 [13.19]	-.04
Tot. Correct_DIST_	-0.05 [0.06]	-.14	<0.01 [0.06]	< .01	0.06 [0.16]	.08	0.13 [0.16]	.17	0.08 [0.16]	.11	0.11 [0.15]	.14
Com. Errs._DIST_	0.04 [0.05]	.17	0.04 [0.05]	.18	-0.16 [0.13]	-.29	-0.17 [0.12]	-.31	0.18 [0.12]	.32	0.14 [0.11]	.25
Latency_DIST_	2.12 [6.75]	.07	-2.43 [6.23]	-.08	-7.26 [16.47]	-.10	-15.14 [15.46]	-.22	21.08 [16.17]	.30	13.92 [14.06]	.20
CR-Score			-.06[0.02]	-.54**			-.10 [0.05]	-.43*			-0.10[0.04]	-.39*
ΔRIIV			-6.36[3.36]	-.29			-16.97 [8.16]	-.33*			-24.63[7.42]	-.49**
*R*^*2*^	.14		.37		.14		.33		.17		.44	
*F*	.85		2.19*		.76		1.71		.99		2.83*	
*ΔR*^*2*^			.23				.19				.27	
*ΔF*			6.04**				4.48*				7.88**	

N = 42. SE = Standard Error. ‘SDQ’ = Strengths and Difficulties Questionnaire; ‘VADPRS’ = Vanderbilt ADHD Diagnostic Parent Rating Scale; ‘Tot. Correct_VIG_’ = Total correct for the Vigilance continuous performance task [CPT]; ‘Comm. Errs._VIG_’ = Commission errors for the Vigilance CPT; ‘Latency_VIG_’ = Latency of correct responses for the Vigilance CPT; ‘Tot. Correct_DIST_’ = Total correct for the Distractibility CPT; ‘Comm. Errs._DIST_’ = Commission errors for the Distractibility CPT; ‘Latency_DIST_’ = Latency of correct responses for the Distractibility CPT; ‘CR-Score’ = Percentage of valid cues correctly reacted to; ‘ΔRIIV’ = cue-detection cost for Residual Intra-Individual Variability.

*p < .05.

**p < .01.

***p < .001.

### Discussion

Correlation analysis between ‘face-valid’ pairs of continuous VMA Task and Continuous Performance Task (CPT) outcomes indicated (in almost all cases) a moderate sized association between outcomes within a pair. The sizes of these associations were on par with the previously reported correlations between corresponding outcomes within these CPTs [83,95], implying that the VMA Task outcomes correlated with corresponding CPT outcomes at least as well as corresponding outcomes within the two CPTs (i.e. Vigilance and Distractibility variants) correlated with one another. As predicted, given the greater methodological similarities, VMA task outcomes showed stronger associations with outcomes from the Distractibility CPT compared to those from the Vigilance CPT format. This is notable because the Distractibility variant of the CPT is intended to be a comparatively more demanding measure of sustained attention than the traditional Vigilance method, which is more sensitive for detecting subtle deficits in this form of attention [84]. Significant inter-assessment agreement was also found to exist for categorical classifications of the number of (i) false reactions on the VMA Task and (ii) commission errors on the Distractibility CPT. This represented a significantly greater probability of participants who made no false reactions also being classified as being within the ‘Normal’ range with respect to commission errors on this CPT. This and the factor structure of the first PCA (Table 7) support the view that false reactions on the VMA task are a valid index of inhibitory control, a facet of executive attention [39,40].

In PCA1 a relationships between VMA task and CPT outcomes were observed within all three extracted factors (Table 7). This suggests convergent validity between these two methodologies in assessing common latent constructs and argues for the feasibility of using more complicated (but potentially informative) visuomotor methodologies like manual tracking to investigate attentional networks [34]. Specifically, Factor 1 suggests that visuomotor control and the extent to which it is impaired is consistent with the relationships seen in the pairwise comparisons. Factor 2 shows associations between response speeds to cues on both tasks, which are also related to an inverse relationship between cue-detection rates and cue-detection costs in tracking (i.e. poorer cue detection associated with longer reaction times but less tracking error). This factor may reflect the strategic components that affect response when visuomotor attention is divided, which were discussed earlier in Experiment 3 (e.g. Loss-Based Selection [71]). It is important to note that the observation of these response patterns in a community-based sample of children is consistent with previous research [72] and argues against attributing similar response strategies in Experiment 3 solely to specific pathological differences between groups with coordination difficulties and those without. Instead, it supports the view that Loss-Based Selection is a context- and ability-dependent strategy employed throughout the lifespan by all individuals independent of impairment [71]. Whether this Factor implicates processing speed (i.e. reaction time) as causal in determining the strategic distribution of resources (slower processing speed creates increased focus on tracking) or an additional by-product of uneven distribution (attentional resources being focussed on tracking creates slower processing speeds) is an interesting future research question. Finally, Factor 3 relates to indices of inhibitory control in both tasks (i.e. false reactions and commission errors) and demonstrates them associating with correct response rates on the CPT task (i.e. less errors relate to more correct responses) and improving with age, a logical pattern given evidence that inhibitory control is an aspect of attention that improves throughout development [60,65].

The second PCA analysis (Table 8) that compared VMA task performance against parental questionnaire responses also supported validation of the VMA task with the finding that two of its outcomes (CR-Score and ΔRIIV) loaded significantly alongside all three questionnaire subscales within the second extracted Factor. Factor 2 loadings suggested this shared variation represented greater ADHD-like behaviours being associated with poorer cue detection rates (CR-Score) but smaller cue-detection costs for participant’s residual intra-individual variability whilst tracking (ΔRIIV).

This relationship with poorer CR-score aligns with previous evidence of children with ADHD showing large impairments on dual-tasks that involve a high degree of motor control [44,96]. Meanwhile, further exploration of the component factors that determine ΔRIIV (i.e. residual intra-individual variability during the first single-target trial and the cue-detection condition) suggested smaller ΔRIIV values were primarily driven by more variance in this outcome during initial single-target tracking (i.e. higher baseline levels of RIIV). Earlier analysis of age group differences in ΔRIIV (Experiment 2) noted a similar pattern of lesser inflation in children’s ΔRIIV when dual-tasking, which was consequently suggested as possibly indicating the extent to which additional executive attentional resources were being recruited to manage visuomotor responses. Finding smaller dual-task costs in this specific component of IIV associating with increased ADHD symptomology concurs with this interpretation because it would again implicate low ΔRIIV as a marker of poorer attentive processing. This might possibly be explained mechanistically by a child requiring additional executive effort to oversee visuomotor control (even when undistracted) and then having reduced additional attentional capacity available in reserve when competing demands emerge during dual tasking.

This interpretation of what ΔRIIV may be a measure of is less consistent with the results of Experiment 3, where ΔRIIV was substantially larger in the coordination difficulties group. However, given the as yet unexplained high degree of co-occurrence for developmental coordination and attentional difficulties [75,80] this perhaps hints at some interesting qualitative distinctions in how attention and motor deficits might differentially affect visuomotor behaviour in demanding environments such as the classroom (i.e. both impair performance but through different mechanistic pathways). To investigate these relationships future research will need to employ more sophisticated modelling techniques (e.g. hierarchical structural equation modelling) to allow more detailed investigation of linkages between attention and action, though this would necessitate substantially larger sample sizes (e.g. n > 400).

Lastly, comparing the predictive validity of this new methodology to pre-existing standardised assessment tools we investigated the extent to which the PCA relationships found between the questionnaires and the VMA task persisted after first controlling for variation explained by age and performance on the continuous performance tasks (Table 9). We observed: (1) none of the existing CPT task outcomes were significant predictors of observational report of ADHD symptomology; (2) for all subscales, inclusion of the VMA task predictors (CR-Score and ΔRIIV) significantly increased the explanatory power of the regression models; and (3) in the case of the SDQ hyperactivity and the VADPRS’s Inattentiveness subscales this resulted in models that explained a significant amount of variation in these outcomes.

In summary, each PCA identified significant overlaps between VMA task outcomes and more traditional methods of assessing attention. This argues for the convergent validity of our novel methodology with existing assessments of childhood attention. Meanwhile, hierarchical linear regression showed that specific outcome measures from the VMA task were predictive of parental report ADHD symptomology even after controlling for variance explained by more traditional assessment methods and age.

## General Discussion

These experiments have demonstrated that: (1) the novel methodology developed for measuring Visumotor Attention (VMA) is sensitive to manipulations of attentional load; (2) there are predictable changes in performance with age and dual task interference, establishing its construct validity; (3) qualitatively different behaviour is observed in children with coordination disorders when assessed using this task; (4) performance on it shares convergent validity with existing standardised psychometric tests of attention and also (5) explains unique variance in validated ADHD screening questionnaire measures, over and above variance explained by existing tests and age.

This is a methodology that holds particular promise as a means of investigating the behavioural integration between attention and action (e.g. in relation to the role the of Loss Based Selection [71] in visuomotor dual-tasking). The computerized format of the VMA task also affords greater objectivity and reliability than more traditional methods, such as pencil-and-paper psychometric tests, individual observation and parent/teacher response questionnaires [7,9]. In addition, it is easier to draw parallels between the manual-tracking responses required on the VMA task and the everyday behaviours required of children in their classroom environments (i.e. it holds face validity for teachers). The VMA task requires participants to produce movements that, in their dynamics (i.e. coupled sinusoidal oscillation), are functionally similar to handwriting and a host of other fine motor manual behaviours [97,98]. Hence, the task assesses how childhood attention functions in educationally relevant scenarios and thus is more grounded in activities of daily living than the abstract methodologies of many existing computerised tests of attention, such as Continuous Performance Tasks (CPTs) [17,18].

Further, the combination of the VMA tasks relatively brief administration time (e.g. faster to deliver than the two CPT subtests in Experiment 4) and the portability of its computerised platform [49,99–101] means that the task has the flexibility to be adapted for use in a range of environments (e.g. it could be administered by clinicians and teachers with minimal training and/or in large-scale community based sampling). Additional advantages include the fact that instructions are delivered by the system (minimizing potential for experimenter bias) and that beyond comprehending and remembering task instructions the memory and prior knowledge demands (e.g. in terms of literacy or numeracy skills) placed on the participant are minimal. In contrast, there are clear contaminating influences of memory related processes in traditional CPT methodologies [102]. This proposed advantage over CPTs is empirically demonstrated in Experiment 4, where CPT performance does not explain significant variation in parental report measures of children’s ADHD symptomology in a community-based sample but specific indices of the VMA task do (viz.: the number of correctly responded cues and residual intra-individual variability in tracking performance across time).

Future research should investigate in more detail intra-individual variability’s (IIV’s) role in tracking performance over time. A relatively rudimentary approach was taken to summarizing IIV in the present studies for expediency (investigating the SD of the overall time-series before and after controlling for shared variability with mean tracking error). Nevertheless, the VMA tool has the capacity to offer far more detailed exploration of this element of response in future studies-something, an element which is particularly difficult to obtain using more traditional point-estimate based computerised methodologies for measuring attentional processes [19,54]. For example, time series of responses have previously been analysed spectrally to identify specific frequency bands more prevalent in individuals with attention difficulties [21,22]. Equally there are alternative summary statistics for describing reaction time variability that take different approaches to controlling outlier responses than the methods we applied [103]. Greater understanding of what IIV signifies is needed because of the acute impact that diagnosed attention disorders are known to have on response variability [21,22,24]. With a task such as the VMA, it is possible to more clearly map fluctuations in attention processes across time in a precise and meaningful manner. This information can then be used to connect attentional control to the external environment and begin to discern principles underpinning this relationship, be they environmental or neurological [34].

Finally, we note that the VMA task shows promise in terms of its psychometric properties. Experiment 3 showed that the outcomes are sensitive measures of group difference caused by clinically significant coordination difficulties and Experiment 4 indicated relationships between certain task outcomes and ADHD symptom report scales. Improving the mechanisms we use to identify and support children with neurodevelopmental disorders is a stated priority within both UK and US public policy [104,105], because of the substantial health, education, social and economic benefits that earlier intervention is predicted to yield (for a review see [106]). Consequently, we propose that the VMA task may have a role to play in more objective population-based screening programmes that aim to chart children’s neuropsychological development as they progress through mainstream education [12].

In conclusion, these findings demonstrate the potential utility of the VMA task for measuring individual differences or examining the effects of interventions on attentional functioning in adults and children (and how these interact with visuomotor control processes to determine behavior). We suggest that VMA provides a means of uniting disparate motor and cognitive accounts of how visuo-manual control is achieved during highly attentionally demanding tasks and thus captures the role that ‘attention’ plays in meaningful everyday behaviour (specifically manual tool use).

## Supporting Information

S1 Dataset Experiment 1 Dataset. Dataset containing the post-processed data for all participants who completed Experiment 1.
(XLSX)

S2 Dataset Experiments 2, 3 & 4 Dataset. Dataset containing the post-processed data for all participants who were recruited to participate in Experiments 2, 3 and 4. Where a participant’s data was not included in the analysis with respect to one of this trio of Experiments (due to differences in their inclusion criteria between experiments) this is identified in this file.
(XLSX)

S1 File Supplementary Analyses. **Additional tables and statistical results.** This file reports the results of additional analysis concerning: (A) post hoc Tukey’s test pairwise comparisons exploring differences in performance due to counterbalance order effects on tracking outcomes in Experiment 1; (B) overall Intra-Individual Variability outcome (IIV) in Experiment 2.
(DOCX)

S1 Text VMA task post-processing guide. A detailed guide of (1) the programmatic steps taken to post-process raw VMA task output into analysable outcome variables and (2) the rationale underpinning the decision-making algorithms developed and applied in this process.
(DOCX)
