# Supplementary material for: Moving to Capture Children’s Attention: Developing a Methodology for Measuring Visuomotor Attention
Source: PLoS One. 2016 Jul 19;11(7):e0159543. doi: 10.1371/journal.pone.0159543 (PMC4951138; doi:10.1371/journal.pone.0159543)
Supplement: S1 File — Additional tables and statistical results. This file reports the results of additional analysis concerning: (A) post hoc Tukey’s test pairwise comparisons exploring differences in performance due to counterbalance order effects on tracking outcomes in Experiment 1; (B) overall Intra-Individual Variability outcome (IIV) in Experiment 2. (DOCX) [file pone.0159543.s003.docx]

**S1. Supplementary Analyses**

**Section A. Experiment 1.**

**Post-hoc Tukey’s Test pairwise comparisons of differences in performance due to counterbalance order for tracking outcomes**

|  | TE (mm^0.5^) | | RIIV (mm^0.5^) | | ∆TE (mm^0.5^) | | ∆IIV (mm^0.5^) | | ∆RIIV (mm^0.5^) | |
| --- | --- | --- | --- | --- | --- | --- | --- | --- | --- | --- |
| Pairwise Contrast | Mean Difference | *p* | Mean Difference | *p* | Mean Difference | *p* | Mean Difference | *p* | Mean Difference | *p* |
| 1st – 2^nd^ | 0.04 [<-0.01, 0.07] | .065 | -0.01 [-0.03, 0.01] | .242 | 0.05 [-0.01, 0.08] | .264 | -0.01 [-0.03, 0.02] | .928 | -0.01 [-0.03, 0.01] | .425 |
| 1^st^ – 3^rd^ | 0.05 [<0.01, 0.09] | .011 | -0.02 [-0.04, -0.01] | .007 | 0.04 [<0.01, 0.10] | .027 | -0.02 [-0.06, <0.01] | .106 | -0.03 [-0.05, -0.01] | .002 |
| 1^st^ – 4^th^ | 0.03 [-0.01, 0.07] | .138 | -0.03 [-0.05, 0.01] | .002 | 0.02 [<-0.01, 0.09] | .085 | -0.03 [-0.06,<-0.01] | .014 | -0.03 [-0.06, 0.01] | .001 |
| 2^nd^ – 3^rd^ | 0.01 [-0.03, 0.05] | .897 | -0.01 [-0.03, 0.01] | .427 | 0.02 [-0.03, 0.06] | .704 | -0.02 [-0.04, 0.01] | .322 | -0.02 [-0.04, 0.02] | .096 |
| 2^nd^ – 4^th^ | -0.01 [-0.04, 0.03] | .985 | -0.01 [-0.03, 0.01] | .230 | 0.01 [-0.04, 0.06] | .939 | -0.03 [-0.05,<-0.01] | .064 | -0.02 [-0.04,<-0.01] | .040 |
| 3^rd^ – 4^th^ | -0.02 [-0.06, 0.02] | .716 | <-0.01 [-0.02, 0.01] | .980 | -0.01 [-0.05, 0.04] | .956 | -0.01 [-0.03, 0.02] | .851 | <-0.01 [-0.02, 0.02] | .986 |

Legend*:* ‘TE’ = Tracking Error; ‘RIIV’ = Residual Intra-Individual Variability; ‘∆TE’ = Cue-detection cost in Tracking Error; ‘∆IIV’ = Cue-detection cost in Intra-Individual Variability; ‘∆RIIV’ = Cue-detection cost in Residual Intra-Individual Variability; *‘*CR-Score’ = percentage of valid cues correctly responded to; ‘Combi.’ = Combined Targets. Values in brackets are 95% Confidence Intervals.

**Section B. Experiment 2.**

**Statistical Analysis of overall Intra-Individual Variability (IIV) outcomes**

For IIV, the MLM results were similar to those already reported for Tracking Error (TE): a main effect of stage, *F*(2,78) = 163.96; *p* < .001, and age group, *F*(1,78) = 128.33; *p* < .001, and a significant interaction, *F*(2,78) = 4.73; *p* = .012). Fig. B1 illustrates this interaction, which was again explored further via analysis of the cue-detection cost for this outcome (∆IIV). For adults, post-hoc pairwise comparisons indicated that performance was significantly worse during the cue-detection condition than for either the first (mean difference [95% CI] = 0.15 mm^0.5^ [0.12, 0.19], *p* <.001) or the second single-target tracking trial (mean difference [95% CI] = 0.13 mm^0.5^ [0.08, 0.18], *p* < .001) but that there was no difference in performance between these two single conditions (*p* = .517). For children, IIV was significantly worse on the cue-detection condition compared to either single-target trial too (ST1: mean difference [95% CI] = 0.20 mm^0.5^ [0.16, 0.25], *p* < .001; ST2: 0.13 mm^0.5^ [0.07, 0.19], *p* < .001) but IIV was also significantly poorer on the second of the two single-target trials than the first (mean difference [95% CI] = 0.08 mm^0.5^ [0.02, 0.13], *p* = .001).

GLM analysis of the cue-detection cost for IIV (i.e. the ∆IIV outcome) showed significant main effects for age-group, *F*(1,76) = 6.30; *p* = .014; η_p_^2^ = .08, and FR category, *F*(1,76) = 7.01; *p* = .010; η_p_^2^ = .08, with no significant interaction (*p =* .702). Estimated marginal means indicated that ∆IIV score was significantly smaller for adults than children (mean difference [95% CI] = 0.05 mm^0.5^ [0.01, 0.09]) and for participants who did not make any false reactions compared with those who did (mean difference [95% CI] = 0.05 mm^0.5^ [0.01, 0.09]).

**

**

**Fig. B1*.*** **Bar graphs depicting the significant interaction between age-group and VMA task stage for Intra-Individual Variability in Experiment 2.** Error bars are 95% confidence intervals.
