# Supplementary material for: Moving to Capture Children’s Attention: Developing a Methodology for Measuring Visuomotor Attention
Source: PLoS One. 2016 Jul 19;11(7):e0159543. doi: 10.1371/journal.pone.0159543 (PMC4951138; doi:10.1371/journal.pone.0159543)
Supplement: S1 Text — A detailed guide of (1) the programmatic steps taken to post-process raw VMA task output into analysable outcome variables and (2) the rationale underpinning the decision-making algorithms developed and applied in this process. (DOCX) [file pone.0159543.s004.docx]

**S1 Text: Supporting Information File**

Post-processing of the Visuo-Motor Attention (VMA) task output

It was necessary to custom process in MATLAB^TM^ (version 7.10.0 R2010a, The MathWorks Inc) the raw output produced for each trial, prior to analysing it. Raw output was initially captured via custom-built software [1] that the VMA task was programmed in. This raw data was then turned into meaningful outcome variables by applying the following sequential steps to post-process it:

1. Converting to a constant sampling rate

KAT software recorded the X and Y co-ordinates of the stylus position on the screen during each trial in all experimental conditions (e.g. single-target, shape, colour and combined), at a sampling rate of *approximately* 120 Hz. The rate was not exactly constant due to fluctuations in the computer’s processing speed though. To achieve a truly constant sampling rate, within each trial we interpolated between discrete data-points to create a continuous time-series for both X and Y on-screen stylus positions. These continuums were then re-sampled at a standardised rate of one reading every 0.83∙ milliseconds (i.e. exactly 120 readings per second). These two new X and Y time-series were then collectively referred to as a trial’s Stylus-position (SP) time-series.

1. Generating Target-position (TP) time-series

To measure tracking performance it was first necessary to create a time-series of X and Y co-ordinates for the movements of the on-screen target(s); so that these could be compared against the stylus position across time. For each of the single-target condition trials this meant one Target-position (TP) time-series, corresponding to the solitary on-screen target. Meanwhile, for cue-detection conditions (i.e. Colour, Shape or Combined), four different TP time-series were generated per trial (i.e. one for each on-screen target). These TP time-series were all generated using the same procedure outline in the next paragraph:

Any given target’s movement pattern within a trial was defined by a sequence of scheduled ‘Events,’ programmed to occur one after the other, for the duration of that trial. Each Event elicited one discrete sinusoidal movement pattern (i.e. the movement ‘paths’ referred to in Experiment 1) that culminated in a return to the original start point. Upon reaching this position the next event was programmed to start^[[1]](#footnote-1)^. The time point at which each Event began was recorded and produced as an additional output alongside the raw X and Y coordinates of the stylus already mentioned (see Section 1).

The type of sinusoidal movement elicited by a specific Event was programmed by manipulating the parameters upon which the dot oscillated along its x and y axes. The factors that were specifiable for each of the waves, independently, were: their start points (*s*) on the respective axes (in X and Y co-ordinates), wavelength (*ʎ*), frequency (*f*) and the phase of one wave to the other. Thus, by combining two different waveforms along the two axes, it was possible to dictate the shape, speed and size of the movement produced during a given Event.

By knowing these parameters for each type of Event (i.e. small Figure-8, Small Boomerang, Large Figure-8 and Large Boomerang) and the start times (t_s_’s) of these Events within a trial (which the software recorded) it was therefore possible to predict the targets’ position at any given time point throughout it. For example, taking a given point in time during a trial (t_z_) this could be cross-referenced against the KAT’s recording of when each Event commenced during that trial, to determine the specific event occurring at t_z_ (e.g. the 5^th^ of the 10 large Figure-8’s). Next, by knowing the movement parameters specified for that type of Event (e.g. the values for *s, ʎ* and *f* ,with respect to both the X and Y axes and the phase relationship between these oscillations) the location of the target (in X,Y coordinates) at t_z_ could be predicted, by applying the following formulae:

X _of target_ = s_X_ + ʎ_X_ × sin((t_z_- t_s_) × *f* _X_)

Y _of target_ = s_Y_ + ʎ_Y_ × sin((t_z_- t_s_) × *f* _Y_)

Thus was it possible to take each time point within the Stylus Position (SP) time-series and predict the corresponding position of each on-screen target at the same point in time.

1. Interpreting Stylus-position (SP) in relation to Target-position (TP)

The multiple TP time-series calculated for a cue-detection condition trial meant that, at any given point during it, there were four potential reference points against which stylus position could be contrasted to estimate tracking performance. Therefore, unlike in the single-target condition, there was no one-to-one correspondence between the stylus and a single on-screen target. Thus, the following additional post-processing of cue-detection condition trials was undertaken:

3.2. The Quadrant time-series (QTS). From a trials’ SP time-series a Quadrant Time-Series (QTS) was extrapolated. This indicated which quadrant of the screen the participant was in at each recorded point in time. Because the four targets’ movement paths were restricted to separate quadrants of the screen, it was logically assumed that if, at a given point in time, the QTS indicated the stylus was in a particular quadrant then the participant was most likely tracking the target bound within that quadrant. For example, if at time (*t*) during a Shape condition trial the QTS indicated the stylus was in the lower left quadrant then it was inferred that the participant was tracking the cross shaped target at that point. Based on this assumption, the QTS could then be used to identify when/if participants made any ‘Switches’ between targets during a trial (represented by a change in quadrant); as well as how long these switches lasted for and between which targets they moved. All such switches of quadrant were provisionally treated as potentially conscious changes in tracking behaviour by the participant (e.g. in reaction to a cue). The validity of this initial assumption was then rigorously assessed by evaluating each switch using the following criteria:

3.3. Interpreting Switches within the QTS. Valid Reaction Windows (VRW’s) within cue-detection trials’ time-series were calculated. These VRW’s were periods of time beginning 0.1 seconds after a valid cue presentation and lasting 2.3 seconds (i.e. until the next [invalid] cue was presented). It was within these time periods alone that it was judged reasonable to expect correct cue responses to have occurred. The timing of these VRW’s in relation to a Switch, combined with the directionality of the Switch (i.e. what quadrants were moved into, out of and through) and its’ timing/directionality in relation to other preceding or following Switches allowed all Switches to be classified as indicative of one or other of the following behaviours (illustrated in Fig. 1):

3.3.1. *Drifting.* This type of behaviour occurred when a participant was tracking a target as it moved close to the edge of its quadrant (i.e. close to the horizontal or vertical midline of the screen). In such instances participants were liable, if they were highly inaccurate in their tracking, to ‘drift’ over the midline for a brief amount of time into one or more of the other quadrants. Whilst this type of movement technically classified as two Switches of quadrant^[[2]](#footnote-2)^ it was unique in having no conscious intention behind it (i.e. the participant was focussed on tracking the same target throughout).

To ensure that such behaviours were not incorrectly interpreted as intentional changes in tracking behaviour any sequence of Switches in quadrant position with a duration of no more than 1.5 seconds and that resulted in a return to the quadrant originally left were edited to indicate the participant had remained tracking the same target throughout the duration of the Drift (i.e. these changes of quadrant were edited-out).

**Fig 1. Diagram illustrating how the quadrant record was used to infer behaviour.** Emboldened ‘quadrant’ sections indicate those currently cued-to. The blue shading indicates Valid Reaction Windows (VRWs) and the red line indicates the values recorded in the Quadrant time-series (QTS) (i.e. which quadrant the stylus is in at each point in time). Notes: The dashed lines do not indicate movement through intervening quadrants (they are merely guide the eye). Also, in practice there was not a fixed interval between each valid cues, this is a simplification made for the diagram (as is the ‘stacking’ the quadrants vertically).

**Legend:**

1) Valid and Correct Reactions

2) Valid but Incorrect Reaction

3) Drifts (< 1.5 seconds long)

4) False Reaction (> 1.5 seconds long)

5) Missed reaction to a cue

6) Indeterminate Movement

3

1

Top Left Quadrant

Top Right Quadrant

Bottom Left Quadrant

Bottom Right Quadrant

3

6

3

1

**Time**

6

2

4

5

The 1.5 second upper limit to define a Drift was chosen because cues were presented at a constant rate of one every 2.3 seconds, each for 0.5 seconds, giving an inter-stimulus interval of 1.8 seconds. Consequently the shortest possible intentional switch between two targets was estimated as being around 1.8 seconds (i.e. the inter-stimulus interval). Assuming a constant reaction and movement time, the briefest conscious response a participant could make would be to (incorrectly) perceive two consecutive cues as valid instructions to move back and forth between two targets (i.e. like a drift); reacting at the last moment of the 1^st^ cue’s presentation and at the first moment of the 2^nd^ cue’s presentation. By choosing the 1.5 seconds limit we set a conservative threshold for classifying drift behaviours, well below the shortest theoretical length of a ‘genuine’ Switch.

**3.3.2.** ***Reactions.*** These were a Switch (or brief sequences of them) that resulted in a change of quadrant for a period greater than 1.5 seconds (i.e. a conscious move to a different quadrant). If such a movement was initiated within a VRW then a ‘Reaction’ to that cue presentation was inferred. Extrapolating from information regarding the time-point at which this Switch occurred during the VRW, a reaction time, movement time and judgement of whether the response was correct (i.e. had they made a ‘Correct’ Reaction?) could then be made (further details later in section 5).

If the Switch had occurred entirely outside any VRW’s then it was inferred to represent a conscious but incorrect decision to change quadrants, most likely indicating the participant having incorrectly responded to an invalid cue (i.e. a black dot). These Switches were recorded as ‘False Reactions.’

**3.3.3.** ***Indeterminate Movements.*** On a small number of occasions participants made a Switch, less than 1.5 seconds in duration, immediately prior to a VRW that then terminated within that VRW, in a different quadrant to the one they initiated from. This behaviour was classified as an ‘Indeterminate Movement’ because it, theoretically, could represent one of two behaviours: (1) a participant being interrupted mid-drift by the cue presentation and responding to it or (2) a ‘False Reaction’ being initiated prior to the cue presentation, followed by a swift response to subsequent cue-presentation. Such behaviour is most likely the former (given the rarity of False Reactions) because this movement occurs in a period where no cues (valid or invalid) are present on the screen. However, the intention behind this specific pattern of movement cannot be as confidently inferred. Furthermore, a Drift (if it is such) seguing into a Reaction obscures the ability to estimate when within the VRW exactly the reaction to the valid cue began (see section 5 later for further explanation). Hence a conservative view was taken that in instances such as these, where the ‘point of reaction’ to a valid cue was ambiguous, it was classified as an un-interpretable period of movement (see Fig. 1 for example).

These ‘Indeterminate Movements’ (IMs) were rare occurrences. At most a participant was recorded as making three of them within a condition (Experiment 1). However, the mean number of IMs made by participants on each of the three cue-detection conditions was 0. There were 48 valid cue presentations per condition which translates to, at worst, 6% of them being un-interpretable. Given the rarity of IMs the small amount of noise they contributed was deemed tolerable.

1. **Generating a time-series of Tracking Errors (TEs).**

The evaluation process described in section 3 resulted in finalised record (the QTS) that classified within a given cue-detection trial: (i) periods of time within its SP time-series that were logically consistent with tracking behaviour, switching behaviour or neither and (ii) at any given time-point the most logical target position (out of the 4 options) to reference stylus position against. Thus it provided the information implicit in all single-target trials (i.e. which target where participants tracking at any given point in time). Subsequently the following metrics of tracking performance could be calculated:

**4.1. Single-target tracking trials.** For this type of trial, the Stylus-position time-series was contrasted against the one available Target-position time-series. For each point in these time series, the straight line distance (in millimetres) between the stylus position and target position coordinates were calculated out using the following formula:

√ (x _stylus_ – x _target_)^2^ + (y _stylus_ – y _target_) ^2^

This time series of distances is referred to hereafter as the time series of Tracking Errors (TEs). It represents the errorfulness of the participants’ tracking behaviour across time (i.e. how far they were away from ‘ideal’ response for each sampled time-point). Note: the first 2.5 seconds of these time-series of TEs were always excluded from any further analysis. This was because this time period always contained abnormally high tracking errors; caused by the participants’ naturally delayed reaction to the initial onset of the dot moving (i.e. the first 2.5 seconds did not denote ‘typical’ tracking behaviour).

**4.2. Cue-detection trials.** Additional steps had to be taken in these dual-task conditions’ trials because of the multiple reference points stylus position could be mapped to at any given time during them (i.e. the four concurrent on-screen target positions). First, the QTS was consulted to identify the most appropriate target to reference the stylus position against at each point in time and then the trigonometric calculations described in section 4.1 were carried out. Next, having obtained a time-series of TEs, sections of it that had earlier been identified as occurring during Reactions (section 3.3.2.) or Indeterminate Movements (3.3.3.) were removed and indexed as missing values. This partitioned off periods of noise within the time-series that did not correspond to true ‘tracking’ behaviour. The first 2.5 seconds of all time-series of TEs was also always excluded (i.e. same as in the single-target condition trials).

**5. Cue-detection outcomes.**

Based on the earlier classifications of quadrant switches against valid reaction windows (VRWs) in section 3.3, the number of correct and false reactions a participant made in each trial could be calculated. A reaction was only counted as fully ‘correct’ if the target the participant had switched to matched the on-screen cue presentation that coincided with this switch. Reactions where a participant had reacted to a valid cue but had inexplicably switched to the wrong target were recorded as ‘valid’ but not correct. Valid reactions were rare and highly correlated with the number of correct reactions participants made, therefore the latter were the only index of valid cue response taken forward for further investigation.

Reaction times for correct and valid reactions were estimable by the following process: switches of quadrant (e.g. like when responding to a cue) created a distinct ‘spike’ in the tracking error (TE) time series at the point they occurred (see Fig. 2).^[[3]](#footnote-3)^ The green ellipse in Fig. 2 highlights this characteristic spike in the time series of tracking error, the upward slope of which is generated by the participant moving across the screen away from one target before (as they switch quadrant), while the downward slope initiates as the stylus positon begins to be referenced against the new target and TE begins to fall rapidly as they hone in on this new target.

Estimated point at which reaction occurs

Switching Quadrant at this point in time

Cue Presentation

**Fig. 2.** **Example of the Tracking Error time-series during a cue reaction**

The largest spike of this nature in a VRW, where a valid/correct reaction was known to have occurred (determined earlier in section 3.3.2), was found by using a peak detection programme written for MATLAB [2]. This spike was then inferred as indicative the reaction (‘switch response’) the participant had initiated. The timing of the first minima of this spike was next found and the difference between that time and the time of the cue presentation was calculated and taken as an estimate of the reaction time (in seconds).

**References**

1. Culmer PR, Levesley MC, Mon-Williams M, Williams JHG. A new tool for assessing human movement: the Kinematic Assessment Tool. J Neurosci Methods. 2009;184: 184–192. doi:10.1016/j.jneumeth.2009.07.025
2. Billauer E. Peakdet: Peak detection using MATLAB*.* 2008. Retrieved 3/3/2014 from: http://www.billauer.co.il/peakdet.html

1. To the participant no discernible break between consecutive Events was apparent because they generated a pattern of continuous smooth movement. [↑](#footnote-ref-1)
2. On occasion can be more than two in a very brief period of time if close to the centre of the screen (the critical feature of this behaviour though is always beginning and ending in the same quadrant) [↑](#footnote-ref-2)
3. This was the reasoning behind differentiating out these switching periods from tracking periods during cue-detection conditions, otherwise measures of tracking error would have been exaggerated by these artefactual spikes (Section 4.2) [↑](#footnote-ref-3)
